# Optimal Allocation of Finite Sampling Capacity in Accumulator Models of Multialternative Decision Making

**DOI:** 10.1111/cogs.13143

**Published:** 2022-05-06

**Authors:** Jorge Ramírez‐Ruiz, Rubén Moreno‐Bote

**Affiliations:** ^1^ Center for Brain and Cognition Department of Information and Communication Technologies Universitat Pompeu Fabra; ^2^ Serra Húnter Fellow Programme Universitat Pompeu Fabra

**Keywords:** Allocation, Accumulators, Decision making, Limited resources, Multialternative, Optimality

## Abstract

When facing many options, we narrow down our focus to very few of them. Although behaviors like this can be a sign of heuristics, they can actually be optimal under limited cognitive resources. Here, we study the problem of how to optimally allocate limited sampling time to multiple options, modeled as accumulators of noisy evidence, to determine the most profitable one. We show that the effective sampling capacity of an agent increases with both available time and the discriminability of the options, and optimal policies undergo a sharp transition as a function of it. For small capacity, it is best to allocate time evenly to exactly five options and to ignore all the others, regardless of the prior distribution of rewards. For large capacities, the optimal number of sampled accumulators grows sublinearly, closely following a power law as a function of capacity for a wide variety of priors. We find that allocating equal times to the sampled accumulators is better than using uneven time allocations. Our work highlights that multialternative decisions are endowed with breadth–depth tradeoffs, demonstrates how their optimal solutions depend on the amount of limited resources and the variability of the environment, and shows that narrowing down to a handful of options is always optimal for small capacities.

## 1 Introduction

The problem of allocating finite resources to determine the best of several options is common in decision making, from deciding which vaccine candidates to fund for further research to choosing a movie for Saturday night. In these cases, planning, and thus resource allocation, needs to be made in advance, well before feedback about the success of the choice is observed. Consequently, two important questions arise: How many options should we examine? And, for how long? When resources are limited, such as number of participants who can be tested with vaccines in a short time, or weekend free time in the previous examples, a decision maker should balance breadth, how many options to sample, and depth, how much to sample each. This ubiquitous decision‐making problem under constrained resources is what has been called the breadth–depth dilemma (Moreno‐Bote, Ramírez‐Ruiz, Drugowitsch, & Hayden, 2020; Horowitz & Sahni, 1978; Miller, 1981).

In the face of many alternatives, humans quickly narrow down the number of considered options to around two to five (Beach, 1993; Hauser & Wernerfelt, 1990; Levin, Jasper, & Forbes, 1998; Olshavsky, 1979; Payne, 1976), and, when presented with more than six options, experienced overload produces suboptimal choices in certain conditions (Iyengar & Lepper, 2000; Scheibehenne, Greifeneder, & Todd, 2010). Models describe this behavior by assuming that considering more options incurs search or mental costs (Hauser & Wernerfelt, 1990; Mehta, Rajiv, & Srinivasan, 2003; Stigler, 1961), but why people consider small sets in a wide range of environments is still a matter of debate. While this could be explained by strict small capacity limits in attention or working memory (Cowan et al., 2005; Miller, 1956), the nature of this small capacity would still need to be addressed (Ma, Husain, & Bays, 2014). Another possibility is that capacity is not necessarily small, but rather that sampling few options and ignoring the vast majority, in either an automatic or in a conscious manner, is actually an optimal policy that favors depth over breadth (Moreno‐Bote et al., 2020). This possibility is supported by the fact that neuronal resources devoted to decision making are not precisely low, as dozens of brain areas and several billions of neurons are involved in even simple decision‐making tasks (Rushworth, Noonan, Boorman, Walton, & Behrens, 2011; Siegel, Buschman, & Miller, 2015; Vickery, Chun, & Lee, 2011; Yoo & Hayden, 2018). Thus, processing bottlenecks could be reflections of close‐to‐optimal policies to breadth–depth dilemmas.

Bounded rationality accounts (Gershman, Horvitz, & Tenenbaum, 2015; Griffiths, Lieder, & Goodman, 2015; Russell & Wefald, 1991; Simon, 1972) surmise that many features of cognition arise from the finite limits of the nervous system. This must also be the case for the nature of the policies chosen by people in decision making, but oftentimes, the constraints imposed by the limited resources are not made explicit. Indeed, choices stemming from sequential sampling between two or three options have been typically modeled as optimal stopping problems (Callaway, Rangel, & Griffiths, 2021; Drugowitsch, Moreno‐Bote, Churchland, Shadlen, & Pouget, 2012; Gold & Shadlen, 2007; Jang, Sharma, & Drugowitsch, 2021; Krajbich & Rangel, 2011; Ratcliff & Murdock, 1976; Sepulveda et al., 2020; Tajima, Drugowitsch, Patel, & Pouget, 2019; Vul, Goodman, Griffiths, & Tenenbaum, 2014), where agents should optimally balance the prospect of learning the value of the options with the costs of sampling them, but they do so without computational or capacity constraints. In these works, the objective is to maximize accumulated reward, typically by introducing a sampling cost. Therefore, by fixating largely on the sequential nature of the tasks, these studies focus only on a particular efficiency–performance tradeoff known as the speed–accuracy tradeoff (Del Giudice & Crespi, 2018). In many complex decisions, however, there are several other functional tradeoffs that involve other properties of the agent–environment loop, such as limited sampling resources, limited interactions with the environment, and delayed feedback (Moreno‐Bote et al., 2020). The effect of these resource limitations on decision making might not be important when there are only two or three available options, but it might be critical when going beyond those low numbers. In that case, the allocation of resources might be governed by two‐stage processes (Hauser & Wernerfelt, 1990; Mehta et al., 2003; Roberts & Lattin, 1991; Shocker, Ben‐Akiva, Boccara, & Nedungadi, 1991), instead of purely sequential processes, where the first decision is about the subset of options that will be considered for further processing.

Here, we study whether narrowing attention to a few options results from optimally allocating finite resources in multialternative choices. To this end, we consider an infinitely divisible sampling resource (e.g., time or precision), such that there are no bounds in the number of alternatives that can be considered. In our model, an agent can first allocate finite sampling time over an arbitrarily large number of options, modeled as accumulators of noisy evidence, with the only restriction that the total sampling time is fixed. This is in stark contrast with previous work on the breadth–depth tradeoff, where the sampling process was simplified, and where the sampling outcomes and resources were discrete, thus obtaining qualitatively different predictions (Moreno‐Bote et al., 2020). This accumulation of evidence runs in parallel and independently for each accumulator, and only their final states are observed. Based on the observations, the agent picks up the one with the highest expected rate of evidence accumulation, which defines the utility of the choice. The goal of the agent is to optimize the allocation of sampling time such that expected utility is maximized. We identify a critical variable in the problem, that we simply call *capacity*, that increases with the actual size of the resources of the agent as well as with the discriminability between options, and we find that this capacity separates two distinct regimes of optimal allocation. When sampling capacity is small, the optimal policy is to sample exactly five options, regardless of the prior. In contrast, when capacity is large, the number of options to sample grows with capacity in a sublinear fashion that depends on the prior. We find a duality between allocated time and allocated precision to the options, such that all our results generalize to allocating precision while keeping fixed sampling time. Finally, we show that even allocations are optimal, and thus better than more complex asymmetric time allocations over the considered options. Overall, our results suggest that decisional bottlenecks can be a byproduct of optimal policies in the face of uncertainty.

**Fig. 1:**
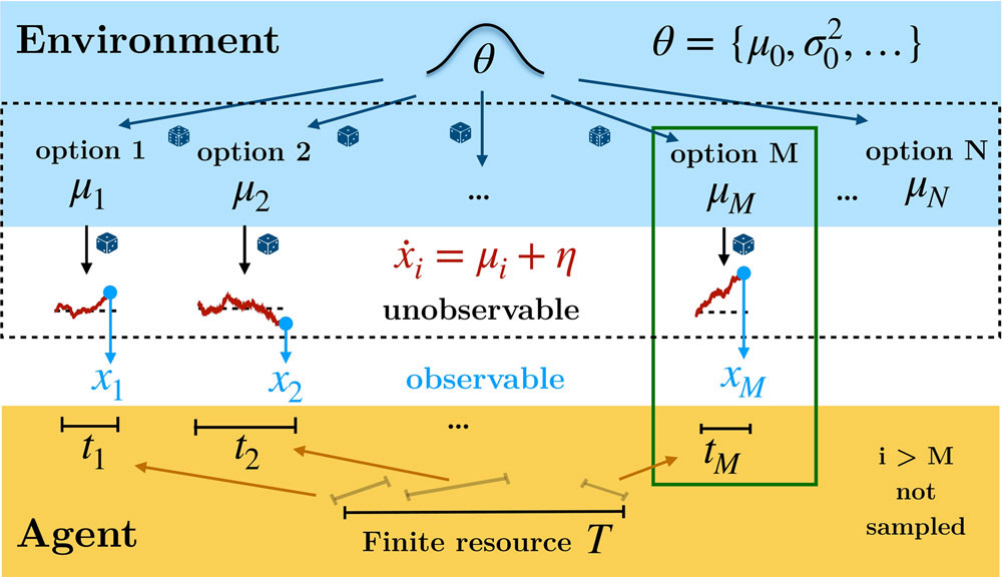
A multiaccumulator model with finite sampling resources. The environment produces a large number of options, each characterized by a drift $\mu_{i}$, unknown to the agent and drawn from a prior distribution characterized by hyperparameters θ, which is known to the agent. The agent has a finite resource T, that they divide and allocate across options, $\sum_{i}t_{i}=T$, in order to sample them. In practice, the agent allocates finite sampling time to a finite number M of accumulators to infer their unknown drifts. After allocation, evidence (red lines) is optimally integrated by the accumulators. The agent observes the integrated evidence $x_{i}$ at the end of the accumulation, after time $t_{i}$, infers the drifts for each of the accumulators and chooses the one that is deemed to have the highest drift (in this case, $\mu_{M}$; green box).

## 2 Multiaccumulator model

We consider an environment that generates many options (N≫1) from which to choose (Fig. 1, top), each one characterized by a ‘drift’ parameter $\mu_{i}$ (i=1,…,N), unknown to the agent. All drifts $\mu_{i}$ are drawn identically and independently from a prior probability distribution $p_{\theta }\left(\mu \right)$, known to the agent and assumed to have finite mean and variance. In order to choose between the options, the agent gathers information by sampling them. The critical aspect of our model is that sampling times $t_{i}\geq 0$ (Fig. 1, bottom) need to be allocated before the actual sampling occurs, and with the constraint that the total sampling time T is limited,

(1) $$\sum_{i=1}^{N}t_{i}=T.$$

In practice, the agent needs to decide on the number of options M≤N to be sampled and their corresponding sampling times $t_{i}>0$ for i≤M, while the remaining options i>M are ignored by giving them no sampling time, $t_{i}=0$ (Fig. 1, bottom). The ordering of the options is irrelevant, as they are initially indistinguishable, and thus we take the first M as those that are sampled. We assume that nonsampled options cannot be chosen, although a ‘default’ option can be added to our framework with no change of our main results.

Once total sampling time is allocated, noisy evidence about the drift $\mu_{i}$ of each of the sampled options i≤M is integrated by independent accumulators (Fig. 1, middle) according to the drift‐diffusion process

(2) $$\frac{dx_{i}\left(t\right)}{dt}=\mu_{i}+\eta_{i}\left(t\right),$$

where $x_{i}\left(t\right)$ is the accumulated evidence up to time t with initial condition $x_{i}\left(0\right)=0$, and $\eta_{i}\left(t\right)$ is a Gaussian white noise with zero mean and fixed variance $\sigma^{2}$, independent and identical for all the accumulators.

The result of the accumulation is the total evidence $x_{i}$ at time $t_{i}$, both of which are observed by the agent and constitute the sufficient statistics for the unknown drift $\mu_{i}$ Moreno‐Bote (2010). With these observations, the agent builds the posterior distribution of the drifts by using Bayes rule as:

(3) $$p\left(\mu_{i}|x_{i},t_{i},\sigma ,\theta \right)=\frac{L\left(\mu_{i}|x_{i},t_{i},\sigma \right)p_{\theta }\left(\mu \right)}{p(x_{i}|t_{i},\sigma ,\theta )},$$

where $L\left(\mu_{i}|x_{i},t_{i},\sigma \right)=N\left(x_{i}|\mu_{i}t_{i},\sigma^{2}t_{i}\right)$ is the likelihood function for the drift, $p_{\theta }\left(\mu \right)$ is the prior distribution, and $p\left(x_{i}|t_{i},\sigma ,\theta \right)=\int d\mu N\left(x_{i}|\mu t_{i},\sigma^{2}t_{i}\right)p_{\theta }\left(\mu \right)$ is the marginal distribution of the evidence, which serves as a normalization constant.

After building these posterior distributions, the agent simply chooses the option with the highest expected drift (Fig. 1, middle, green box), which defines utility, $U\left(M,x,t,\sigma ,\theta \right)=\max_{i\leq M}{\hat{\mu }}_{i}\left(x_{i},t_{i},\sigma ,\theta \right)$, where $x=(x_{1},\text{…},x_{M})$ is the vector of observations for the M accumulators with allocated times $t=(t_{1},\text{…},t_{M})$. To avoid notation clutter, from now on, we will stop writing the dependence on σ and θ of the various functions and leave it implicit.

The previous expression is the utility of the choice of accumulator, which depends on the observations and allocation times. However, before time is allocated, the observations x themselves will be unknown to the agent. Therefore, the expected utility of a given allocation t is given by taking the expectation of the above utility over all possible observations as:

(4) $$\hat{U}\left(M,t\right)\equiv E\left[\max_{i\leq M}{\hat{\mu }}_{i}|t\right]=\int dx_{1}\text{…}dx_{M}p\left(x_{1},\text{…},x_{M}|t\right)\max_{i}{\hat{\mu }}_{i}\left(x_{i},t_{i}\right),$$

where, using the independence of the accumulators, $p\left(x_{1},\text{…},x_{M}|t\right)=\prod_{k\leq M}p\left(x_{k}|t_{k}\right)$ is the product of the marginal distribution of the evidence.

Optimally inferring the drifts from observations is readily accessible through Bayesian inference as shown above. Thus, the main, and harder, objective of the agent is to optimize the allocation policy, that is, to select both the number of sampled accumulators M≤N and the time $t_{i}$ allocated to each, in order to maximize expected reward, while satisfying the total sampling time constraint in Eq. 1. This is accomplished by optimizing the utility with respect to M and $t=(t_{1},\text{…},t_{M})$ as:

(5) $$\left(M^{*},t^{*}\right)=\arg\max_{M,t}\hat{U}\left(M,t\right).$$

## 3 Capacity and time–precision duality

**Fig. 2:**
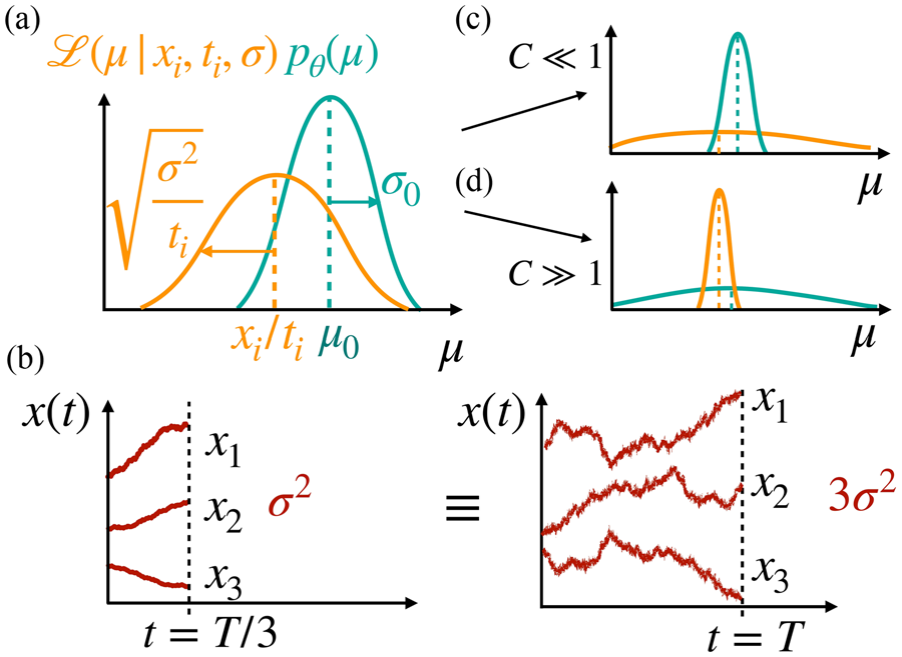
Time/precision duality and the notion of capacity. (a) The likelihood of the drift μ (in orange) given the evidence has variance $\sigma^{2}/t_{i}$ and the prior distribution of the drifts (in cyan) has variance $\sigma_{0}^{2}$. These quantities determine capacity as in Eq. 6. (b) Time and sampling noise are intricately related (see text). In this example, allocating time T/3 to each accumulator under fixed precision $1/\sigma^{2}$ (left) is equivalent to allocating precision $1/3\sigma^{2}$ to each accumulator under fixed sampling time T (right). (c) Small capacity means that the variance of the observation is much larger than the variance of the prior, indicating that it is difficult to confidently identify the best drift from the observations. (d) In the large capacity limit, it is easier to differentiate the good drifts from the poor ones.

While time can be understood as the resource that the agent allocates, we found a dimensionless scale that expresses their actual sampling capacity, that is, their ability to sample and differentiate between drifts, which we call capacity C (Fig. 2). As the agent integrates noisy evidence through Eq. 2, the likelihood of the drift $\mu_{i}$ for accumulator i is proportional to a Gaussian (Fig. 2a, orange curve) with mean $x_{i}/t_{i}$ and variance $\sigma^{2}/t_{i}$, $L\left(\mu_{i}|x_{i},t_{i},\sigma \right)\propto N\left(\mu_{i}|\frac{x_{i}}{t_{i}}\frac{\sigma^{2}}{t_{i}}\right)$ Moreno‐Bote (2010). Its variance $\sigma^{2}/t_{i}$ shows how the sampling time and the variance of the sampling noise are related when inferring the drift $\mu_{i}$: the likelihood gets broader with increasing σ or decreasing time $t_{i}$, reflecting that the precision of the observations is decreased by having more noise or less time, respectively. In fact, the sampling capacity of the agent should capture this duality. Thus, having a fixed capacity could be interpreted as having a fixed noise variance $\sigma^{2}$ for all accumulators and allocating time T between them (Fig. 2b, left) or as having a fixed sampling time T for each of the accumulators and allocating precision $1/\sigma^{2}$ between them (Fig. 2b, right).

Moreover, the posterior in Eq. 3 depends on the prior as well (Fig. 2a, cyan curve). For fixed evidence, the broader the prior is, the easier it is to differentiate between sampled drifts, since the expected squared distance between two drifts drawn from the same distribution is twice its variance $\mathrm{Var}[p_{\theta }\left(\mu \right)]$ (see online Appendix A.1). Therefore, we define the capacity allocated to option i as the ratio between the precision of the observation and the precision of the prior,

(6) $$c_{i}=\frac{\mathrm{Var}[p_{\theta }\left(\mu \right)]}{\mathrm{Var}\left[N\left(\mu_{i}|\frac{x_{i}}{t_{i}},\frac{\sigma^{2}}{t_{i}}\right)\right]}=\frac{\sigma_{0}^{2}}{\sigma^{2}}t_{i}.$$

Adding the individual capacities results in the total sampling capacity of the agent,

(7) $$C=\sum_{i}c_{i}=\frac{\sigma_{0}^{2}}{\sigma^{2}}T.$$

For the rest of this article, we stick to the interpretation of allocating capacity as dividing the total time T while fixing the accumulation noise σ, such that the variable we can control is the sampling time allocated to each option, keeping in mind that all the results presented below can be readily reinterpreted as dividing precision while giving to all options the same sampling time.

## 4 Results

Optimally dividing sampling capacity C into options is an a priori hard problem due to its high dimensionality. However, we show in Subsection 4.2 that the optimal allocation lies within the family of even allocations, where M options receive equal sampling time $t_{i}=t\equiv T/M$, while the remaining others are given no time. Thus, finding the optimal policy reduces to finding the optimal number M of accumulators to sample.

### 4.1 Even sampling

In this subsection, we exploit the structure of even sampling. First, the posterior mean of the drift ${\hat{\mu }}_{i}\left(x_{i},t\right)$, computed from Eq. 3, is a monotonously increasing function of the evidence $x_{i}$ for any prior (see proof in Section A.2 in online Appendix). Therefore, the option that maximizes the posterior mean ${\hat{\mu }}_{i}$ is the one that has the highest evidence $x_{i}\left(t\right)$, as all M sampled options are given the same sampling time t. This allows us to work by maximizing evidence instead of maximizing the posterior means of the drifts in Eq. 4. Second, by changing variables $y\equiv \max_{i}x_{i}$, and using the probability distribution of the maximum y, denoted by $p_{\max}\left(y|t,\sigma ,\theta \right)$, the expected utility in Eq. 4 can be recast in the one‐dimensional integral

$$\hat{U}\left(M,t\right)=\int \mathrm{dy}p_{\max}\left(y|t\right)\hat{\mu }\left(y,t\right).$$

Finally, given that the M options are sampled evenly, the probability distribution of the maximum can be simplified by using the cumulative distribution of the evidence x for an arbitrary accumulator, $F_{x}\left(y|t\right)=\int_{-\infty }^{y}dx^{\prime }p\left(x^{\prime }|t\right)$, where p(x|t) is the marginal of the evidence x of the accumulator, as:

(8) $$p_{\max}\left(y|t\right)=\frac{d}{dy}{\left[F_{x}\left(y|t\right)\right]}^{M}.$$

With all the above, the expected utility in Eq. 4 can thus be written as:

(9) $$\hat{U}\left(M,t\right)=M\int \mathrm{dy}{\left[F_{x}\left(y|t\right)\right]}^{M-1}p\left(y|t\right)\hat{\mu }\left(y,t\right).$$

When the prior distribution is a Gaussian with mean $\mu_{0}$ and variance $\sigma_{0}^{2}$, it is possible to identify the total capacity $C=\frac{\sigma_{0}^{2}}{\sigma^{2}}T$ explicitly and Eq. 9 simplifies to

(10) $$\hat{U}\left(M,C\right)=\mu_{0}+\frac{M\sigma_{0}}{\sqrt{1+\frac{M}{C}}}\int_{-\infty }^{\infty }\mathrm{dy}{\left[\Phi (y)\right]}^{M-1}N\left(y|0,1\right)y,$$

where $\Phi \left(y\right)=\frac{1}{2}\left[1+erf\left(\frac{y}{\sqrt{2}}\right)\right]$ is the cumulative distribution function of a normal distribution.

**Fig. 3:**
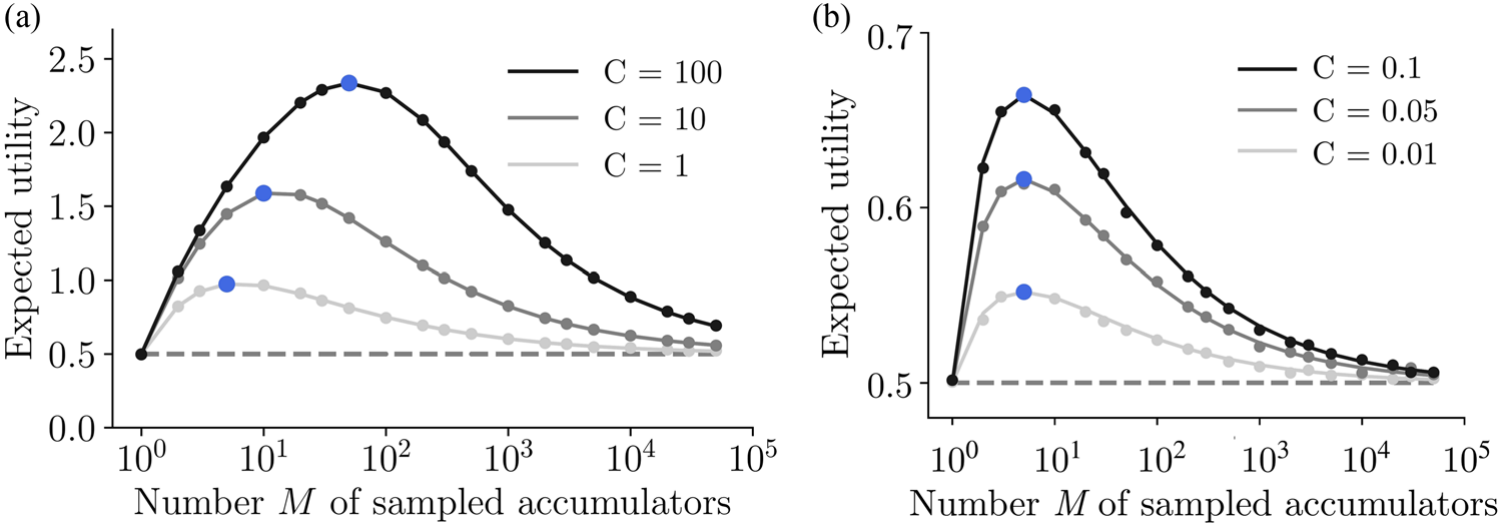
Expected utility as a function of sampled accumulators exhibits the breadth–depth tradeoff. Results for the Gaussian prior case ($\mu_{0}=0.5,\sigma_{0}^{2}=1$), for various different capacities. Blue points denote the maxima. Note log horizontal scale (points, Monte Carlo simulations; lines, theoretical predictions, Eq. 10). (a) For large capacities, the optimal number of sampled accumulators changes with the capacity. (b) For small capacities, the optimum is independent of capacity and equal to five.

Plotting the utility in Eq. 10 as a function of the number of sampled accumulators M reveals a clear breadth–depth tradeoff (Fig. 3a). At the depth limit, M=1, only one accumulator is sampled and it is given all sampling time T. In this case, the expected utility will simply be the expected value of the prior, $\mu_{0}=0.5$ (Fig. 3a, left point), since there is no choice to be made between accumulators. At the breadth extreme, M/C→∞, the evidence gathered for each accumulator is very noisy because each has been allocated a very short sampling time, and thus choosing any will amount to an expected utility again equal to the prior mean (rightmost points). Therefore, for all capacities, there is an intermediate optimal value for the number of accumulators to sample, $M^{*}$.

#### 4.1.1 Sharp transition between the small and large capacity regimes

Our main result is that the optimal allocation policies are qualitatively different at small and large capacity, and that there is a abrupt transition between the two regimes. We provide useful asymptotic analytical expressions for the utility in Eq. 9 and the optimal $M^{*}$ in both limits and describe their characteristic features.

The limit C≪1 corresponds to the case where the uncertainty in the observation $\sigma^{2}/T$ is much larger than the variance of the prior $\sigma_{0}^{2}$, that is, the Gaussian likelihood is much wider than the prior (Fig. 2c). In this limit, we find that the utility in Eq. 9 can be expanded a series in powers of $\sqrt{C}$, which at first order is given by (see online Appendix, Section A.3)

(11) $$\hat{U}\left(M,C\right)=\mu_{0}+\sigma_{0}\sqrt{\frac{C}{2\pi }}\left[\sqrt{M}\int_{-\infty }^{\infty }dzz\exp\left(-\frac{z^{2}}{2}\right){\left(\frac{1}{2}+\frac{1}{2}erf\left(\frac{z}{\sqrt{2}}\right)\right)}^{M-1}\right]+O\left(C\right).$$

**Fig. 4:**
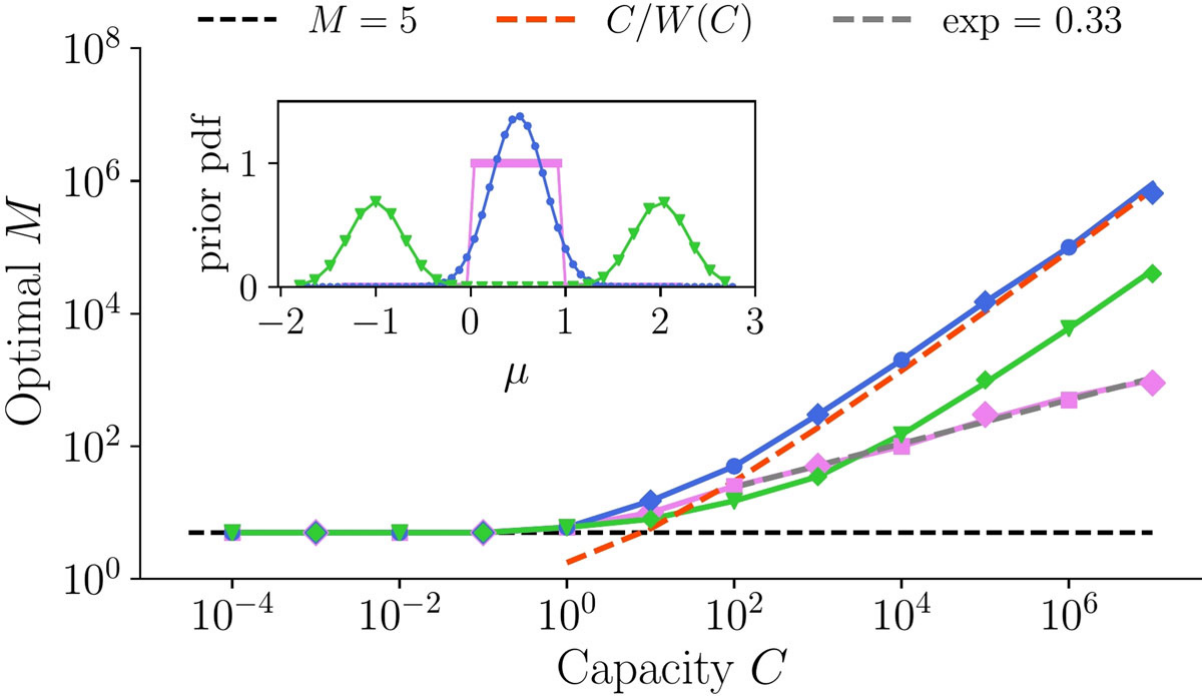
The optimal number of sampled accumulators undergoes qualitatively different behaviors at small and large capacity values. Results come from searching the maximum expected utility via Monte Carlo simulations (points) and numerical integration (lines) for a Gaussian (blue line; Eq. 10), uniform (pink; Eq. A.6), and a bimodal (green; Eq. A.7) priors (illustrated in inset). We used $\mu_{0}=0.5$ for all priors and $\sigma_{0}^{2}=1/12$ for the Gaussian prior to match the uniform distribution. For the bimodal prior, the variance of each mode equals $\sigma_{0}^{2}$. Dashed red line corresponds to the asymptotic limit in the Gaussian prior case, Eq. 13. Dashed gray line (almost overlaid by the pink line) is the best power law fit for the uniform prior case ($M^{*}\propto C^{a},a=0.33$). Diamonds (overlaying most of points) indicate simulations with a ‘default’ option.

Remarkably, this expression holds for any prior distribution as long as capacity is small enough. Let us now note that the only dependence on M appears in the quantity in square brackets, so we isolate it to look for the optimal M. Since capacity does not play a role here, we see that $M^{*}$ will be constant as a function of small capacity. Furthermore, using Extreme Value Theory (see Section A.5 in online Appendix), we find that this quantity decreases with M for large M. This means that sampling many accumulators will not be optimal, following the intuition that there is no point in sampling many options when having scarce resources. On the other hand, and as noted before, it is easy to see that expected utility attains its lowest value when M=1, since in this case, there is no choice to be made. Thus, the optimal M is attained at some intermediate value. Given these observations, the optimum can be thus found numerically by varying *M*, and it happens when

$$M^{*}\left(C\ll 1\right)=5,$$

which can be checked visually for various values of small capacity in Fig. 3b, which also validates the approximation in Eq. 11. It is important to highlight that when capacity is strictly zero, expected utility does not depend on M, and is equal to the prior mean, since choosing options to sample has no effect when there is no time to be allocated. However, as long as the small capacity is finite, an optimal number of options to sample equal to five emerges, regardless of the prior and the value of capacity. We have confirmed this strong prediction by direct numerical integration of Eq. 9 using different prior distributions, including Gaussian, uniform, and bimodal (Fig. 4), which also holds even when a nonsampled, default, option can be chosen (diamond markers).

The opposite limit C≫1 corresponds to the case where the precision of the observation is much greater than the one of the prior (Fig. 2d). Intuitively, this means that the quality of the observations is good enough to likely differentiate the drifts between two randomly chosen accumulators, and thus we expect the optimal number of accumulators to increase with increasing capacity, giving a qualitatively different behavior than at small capacity. Hence, we make this assumption to inspect the optimality of Eq. 9 for this large capacity limit, which we find to be consistent with the numerical results shown below. In particular, when the prior distribution is Gaussian, the expected utility in Eq. 10 has the following asymptotic behavior,

(12) $$\hat{U}\left(M\gg 1,C\right)\to \mu_{0}+\sigma_{0}\frac{b_{M}}{\sqrt{1+\frac{M}{C}}},$$

where $b_{M}={\left(2\log\left(M\right)-\log\left(\log\left(M\right)\right)-\log\left(4\pi \right)\right)}^{1/2}$ (see Section A.5 in online Appendix). By relaxing M to be continuous, we can maximize expected utility, and we find that the optimal number of sampled options for large capacity satisfies, up to leading order, the implicit equation $M^{*}\log\left(M^{*}\right)=C$. After inverting it, the optimal number of sampled options is

(13) $$M^{*}\left(C\gg 1\right)=\frac{C}{W(C)},$$

where W(C) is the Lambert function. This asymptotic limit provides a very good approximation to the optimal $M^{*}$ at large C obtained from direct numerical integration of Eq. 10 (Fig. 4; red dashed line, theory; blue points, simulations). For prior distributions other than the Gaussian, we rely on numerical integration of Eq. 9 (see Sections A.6 and A.7 in online Appendix for analytical expressions). For a uniform prior, the optimal number of sampled options increases as a power law with an exponent close to 1/3 (Fig. 4, pink), while for a bimodal prior, the optimal number increases in a similar fashion to the Gaussian prior case (green). While differences of asymptotic limits are due to the presence of bounded or unbounded drifts in the priors, in all cases, the increase is sublinear, indicating that increasingly longer times are allocated to each of the sampled accumulators as capacity increases.

The above results show that there are two distinct regimes, one at small and another at large capacities, characterized by qualitatively different optimal allocations: while at small capacity, the optimal number of sampled accumulators should be five regardless of the prior, at large capacity, the optimal number of sampled accumulators grows sublinearly regardless of the tested prior. Further, we observe that there is an abrupt transition between the two regimes as capacity grows, with a bump being observed at intermediate capacity values.

### 4.2 Even allocation is optimal

Above, we have assumed that we could find the optimal time allocation within the subset of even allocations, such that, given finite total time T, an agent just needs to determine how many options will be sampled and split equal time to all of them. Conveniently, this set is discrete and thus amenable to effective search of the optimum. However, in general, the set of allocation policies is the infinite‐dimensional simplex $\sum_{i}t_{i}=T$, $t_{i}\geq 0$ for all i, as a priori the agent could unevenly split time to options in any arbitrary way. Despite its infinite‐dimensionality, we have seen in the case of even sampling that it is optimal to ignore (infinitely) many options, such that $t_{i}>0$ only for i∈{1,…,M}, with finite M, to which we will refer as having M active dimensions.

To address the most general case, using the above intuitions, we first generalize the expected utility, Eq. 9, to the case when allocated time is unevenly distributed among M accumulators, as:

(14) $$\hat{U}\left(M,t\right)=\int_{-\infty }^{\infty }dy\frac{d}{dy}\left[\prod_{i=1}^{M}F_{x}\left(y|t_{i}\right)\right]\hat{\mu }\left(y,t_{i}\right),$$

where $F_{x}\left(y|t_{i}\right)$ is the cumulative distribution function of the posterior when using $t_{i}$ sampling time. Our goal is then, for every M, to find the allocation t that maximizes Eq. 14 under the capacity equality constraint and the inequalities $t_{i}\geq 0$ for all i, and then select the optimal M, the one that achieves the highest utility.

In this more general setup, an even allocation corresponds to the symmetrical point in M active dimensions given by $t_{M}^{e}$, where $t_{M,i}^{e}=T/M$ for i=1,…,M (superscript reflects ‘even’ allocation). As the expected utility in Eq. 14 is symmetric under any permutation $t_{j}↔t_{k}$ for any j and k, all its partial derivatives have to be equal at $t_{M}^{e}$. Therefore, every even allocation for each M corresponds to a critical point of the constrained optimization problem (see online Appendix, Section A.8).

**Fig. 5:**
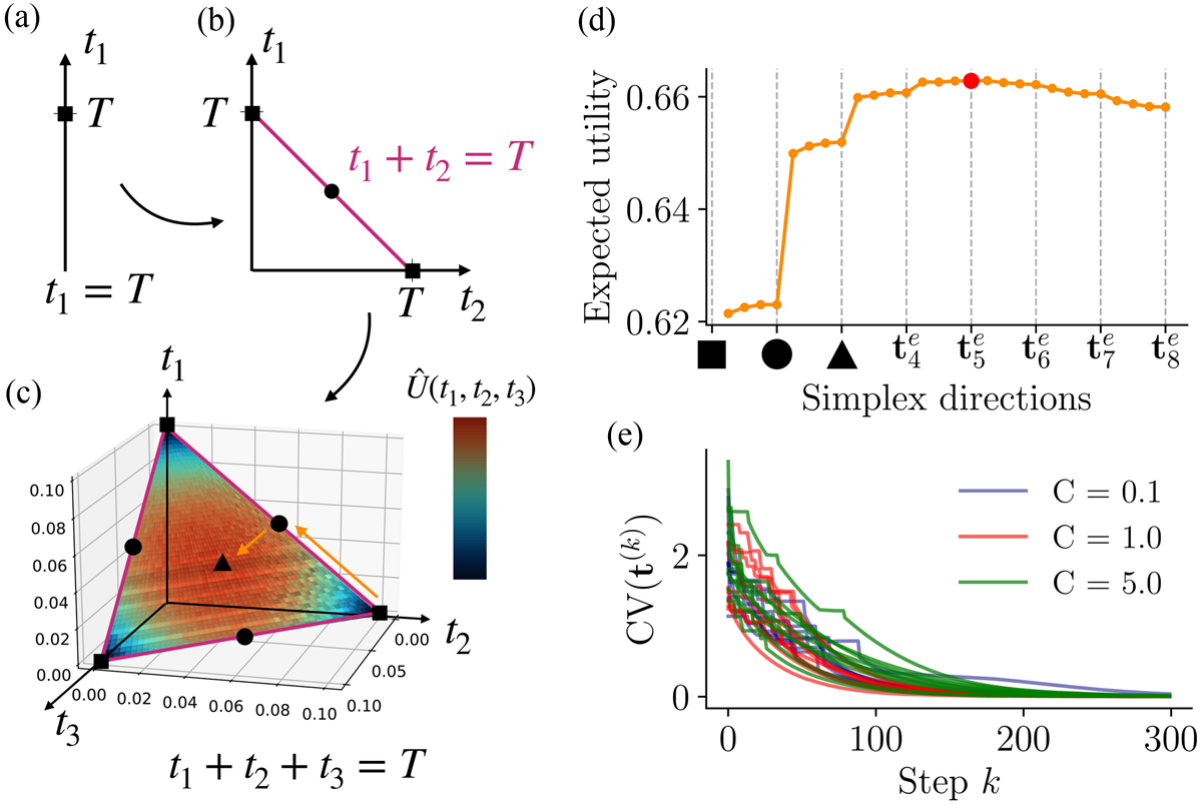
Even allocations correspond to critical points of utility lying at the center of M‐simplices. (a) In one dimension, there is only one point that complies with the constraint. (b) For M=2 dimensions, constraints define a line segment or 1‐simplex. The circle depicts the symmetric critical point $t_{2}^{e}$. (c) For M=3, constraints form a triangle or 2‐simplex. The black triangle is the symmetric critical point $t_{3}^{e}$. The colors at the extremes reflect the minimum and maximum utility reached in this simplex, which was computed with Monte Carlo simulations of Eq. 4 for the Gaussian prior with T=0.1, σ=1, $\sigma_{0}=1$, $\mu_{0}=0.5$. (d) Expected utility computed along directions that go orthogonally from $t_{M}^{e}$ to $t_{M+1}^{e}$ (as illustrated with orange arrows in panel c, same parameters). The red dot shows the maximum occurring at $t_{5}^{e}$. (e) Using the stochastic projected gradient ascent detailed in Section A.8, we initialized the algorithm at random points (10 shown here) in a high‐dimensional simplex and measured the coefficient of variation (CV) of the allocation vector at every step of the algorithm until convergence, for various values of capacity. Zero CV implies even allocation.

We still need to characterize these critical points in order to show that the global maximum is indeed an even allocation. We first remember that the optimal number of active dimensions M needs to be found, and thus it is useful to see how expected utility varies as a function of M. To do this, we note that any M‐dimensional simplex is in fact the border of an (M+1)‐dimensional simplex. For example, for M=2, the constraints describe a line segment, or 1‐simplex, where we have the symmetric critical point $t_{2}^{e}=\left(T/2,T/2\right)$ (Fig. 5b, black circle). We then notice that the line $t_{1}+t_{2}=T$ is one of the three edges of the triangle, or 2‐simplex (Fig. 5c: pink lines are the edges of triangle), where in fact we have another symmetric critical point in its interior (black triangle). With this, we can ‘visualize’ the infinite‐dimensional nature of this problem, since all critical points of the utility lie at the edges of a higher dimensional simplex.

To assess the landscape of expected utility in high‐dimensional simplices, we can evaluate it at all symmetric critical points $t_{M}^{e}$ and along directions that go orthogonally between them (Fig. 5c, orange arrows). Thus, we devised a one‐dimensional path that allows to continuously connect all symmetrical critical points, and applied it to the small capacity limit C≪1. As we move from the 1‐simplex to higher dimensional simplices (as in Fig. 5c), we find that first utility increases, reaching a maximum at the even allocation in M=5 dimensions, and then decreases (Fig. 5d). Therefore, critical points $t_{2}^{e}$, $t_{3}^{e}$, and $t_{4}^{e}$ are ‘saddle'‐like points, as they are maxima in the interior of their corresponding simplex, and minima as one moves to the interior of the higher dimensional simplex.

Although the above analysis suggests that the optimum lies at an even allocation point, it is still unclear whether there are other critical points that are asymmetrical and have a larger utility. To argue that the presence of nonsymmetrical local optima is unlikely, we used a stochastic gradient projection method (Fletcher, 2013) that maximizes expected utility subject to the constraints, and applied it to the Gaussian prior case (see Section A.8 in online Appendix for details). Indeed, we find for various capacities that, regardless of the initial condition, that is, random initial allocations, a maximum utility is attained when time is evenly divided (Fig. 5e), and the global maxima coincide with the ones found in the previous sections.

## 5 Discussion

We have studied a model of multialternative decision making where an agent can allocate finite sampling resources to options and choose the best one among them. We found that the capacity of the agent depends on both the amount of sampling resources, that is, time or precision, as well as on the discriminability of the options in the environment. As a function of capacity, optimal policies undergo an abrupt transition: at small capacity, allocating time to a handful of options is optimal; at large capacity, the number of options grows sublinearly, well below the actual sampling capacity of the agent. Our results show that decision bottlenecks, such as option‐narrowing, can arise from optimal policies in the face of uncertainty, and provide so far untested predictions on choice behaviors in multialternative decision making as a function of capacity.

Seemingly strict limits pervade cognition, from the so‐called attentional bottleneck (Deutsch & Deutsch, 1963; Treisman, 1969; Yantis & Johnston, 1990), over working memory (Brady, Konkle, & Alvarez, 2011; Cowan et al., 2005; Luck & Vogel, 2013; Miller, 1956; Ma et al., 2014), to executive control (Norman & Shallice, 1986; Shenhav et al., 2017; Sleezer, Castagno, & Hayden, 2016). These limits might result from using scarce neuronal resources or from using them inefficiently. However, a likely alternative is that bottlenecks reflect strategies that make optimal use of limited but large resources. Indeed, past work has recognized that some apparent limits, most notably dual tasking bottlenecks (Fischer & Plessow, 2015; Meyer & Kieras, 1997), could be the result of optimal allocation of finite resources to avoid overlap and interference between the different representations needed to solve the two tasks (Feng, Schwemmer, Gershman, & Cohen, 2014; Meyer & Kieras, 1997; Zylberberg, Dehaene, Roelfsema, & Sigman, 2011). Further, it has been recognized that the narrow focus of attention could be at the heart of solution to the binding problem by integrating separate features into a coherent object (Treisman, 1998), and thus, its narrowness might reflect a function more than a limitation. Our work follows this line of argument and provides for the first time a quantitative account for why it is optimal for an agent to consider a handful of options in the face of uncertainty, well above two but well below 10. In addition, our results shed light on why people might ignore hundreds of accessible options and focus resources to a very small number of options (Hauser & Wernerfelt, 1990; Iyengar & Lepper, 2000; Scheibehenne et al., 2010). Thus, some of the seemingly strict limits in decision making can be the result of optimal policies that favor depth versus breadth processing of the options.

It has been long recognized that people often consider a small set of options while ignoring many others (Hauser & Wernerfelt, 1990; Mehta et al., 2003; Payne, 1976; Stigler, 1961). In the ‘consumer’ literature, this is explained by arguing that small consideration sets are favored because they optimally balance the probability of finding a good option in the set with the search and mental costs incurred in adding new options to that set. These models thus assume that resources are not limited, but are costly. In contrast, the assumptions in our work do not explicitly tune the cost of sampling, but rather an implicit cost arises naturally from the strict capacity constraint, which depends intrinsically on the agent as well as extrinsically on the environment. A more fundamental distinction is that previous work did not focus on allocating resources intensively into the options, such that the only decision was whether to include an option into the set or not, without considering the amount of resources allocated to it. This distinction makes that problem drastically different than the tradeoffs of the breadth–depth dilemma considered here. This can explain why transitions of optimal policies as a function of agent's parameters have not been reported before.

Most current theories of perceptual and value‐based decision making are based on accumulation of evidence that favors certain hypotheses over others (Drugowitsch et al., 2012; Gold & Shadlen, 2007; Moreno‐Bote, 2010; Ratcliff & Smith, 2004). When combined with sampling costs and rewards, a normative theory emerges where it is optimal to only accumulate evidence up to a bound (Drugowitsch et al., 2012; Gold & Shadlen, 2007). By letting the information between the competing options be processed parallelly, a tradeoff between speed and accuracy of the choice emerges, and it is then possible to derive optimal policies under various further assumptions (Callaway et al., 2021; Jang et al., 2021; Tajima et al., 2019; Vul et al., 2014). For instance, the work of Vul and colleagues assumes that only one option is correct, that is, that there are only two types of reward. Combined with an opportunity cost, taking zero to one sample in this setting becomes optimal under large parameter regions of the cost of time (Vul et al., 2014). However, multialternative decision making requires estimating the subjective value of offers, and none of them is correct in any absolute sense, which can favor not single‐sample, deep strategies (Moreno‐Bote et al., 2020). The work of Jang and colleagues aims to optimally allocate attentional resources by solving numerically the Bellman equation in binary choice (Jang et al., 2021), but extending this framework to (many) multialternative decisions is intractable. While the speed–accuracy tradeoff is a ubiquitous phenomenon in sequential decisions, there are certainly other features of decision making that need to be studied in isolation to advance our understanding of the various challenges that arise in multialternative decision making.

In our work, we have implemented the prevalent feature of evidence accumulation, but we have highlighted other characteristics of decision making, such as finite sampling resources, delayed feedback, and limited interaction with the environment, that are critical in many real‐world examples (see Introduction). These assumptions differentiate our results with those of previous work. Most importantly, we have not considered a sequential process where the evidence gathered during the accumulation is observable and thus, it cannot be used to stop the accumulation process. This is an important case when accumulation of evidence happens in a decentralized manner by, for example, different groups of neurons, and only the final result of the accumulation is conveyed to another set of neurons where the comparison and choice takes place. Indeed, parallel sampling of information is a ubiquitous ingredient in theories of decision making (Busemeyer & Townsend, 1993; Glöckner & Betsch, 2008). In this work, limited capacity in parallel sampling is understood as a limitation on available sampling precision, thus allowing for the allocation of attentional resources under parallel evidence accumulation with time pressure (Fig. 2). These ingredients force the decision maker, in a deliberate or automatic way, to allocate resources into the options in advance in a strategic fashion, prompting the need to trade sampling breadth over depth.

Although previous work has characterized optimal breadth–depth tradeoffs in multialternative choices like the ones studied here, it has been assumed that agents have a finite ‘discrete’ capacity (Moreno‐Bote et al., 2020). Our assumption of a continuous resource (e.g., time or precision) that can be infinitely divided has allowed us to uncover qualitatively novel optimal policies at small capacity. We have, therefore, been able to derive optimal policies that trade off breadth with depth search in (many) multialternative settings where using traditional sequential decision frameworks would be intractable. Integrating resource allocation with sequential decision making into a single theory of dynamic allocation will be most relevant to understand human decision making, but its study is deferred to the near future. In any event, any agent with finite capacity cannot avoid the problem of first deciding how many options to allocate capacity to, and thus breadth–depth tradeoffs as described above will be generally at play.

Bounded rationality accounts (Gershman et al., 2015; Griffiths et al., 2015; Russell & Wefald, 1991; Simon, 1972) propose that cognition results from the finite limits of the nervous system from where it emerges. Our work follows this line of research in two ways. First, we propose that agents indeed have a finite sampling capacity that can be arbitrarily allocated to the available options. However, an important assumption in our work is that while the intrinsic resources of an agent might seem large, the interaction of the agent with the environment might render their effective decision‐making capacity small. Therefore, capacity is not an absolute quantity that describes an agent, but a relative quantity that contextualizes the agents and characterizes how well they are suited to solve a given task in the world. An important contribution of our work is to show that optimal policies depend on effective capacity in a highly nonlinear way, such that small‐capacity agents would behave qualitatively different than large‐capacity agents (or even the behavior of the same agent operating in different environments could be qualitatively different). This is clearly a prediction that can be tested with humans where time or other resources are constrained and varied on a trial by trial basis. Second, agents perform the allocation before feedback is received, which relates to a bounded‐optimal agent that is optimized at ‘design'‐time, which eliminates the paradox of perfect rationality by not letting the agent optimize their decisions at run‐time (Russell & Subramanian, 1994), an argument that further supports the validity and relevance of two‐stage decisions.

Another important result of our work is that evenly dividing time to a small set of options is optimal when they are initially indistinguishable. This optimal division of resources coincides with the 1/N heuristic rule (Gigerenzer & Gaissmaier, 2011) or equality heuristic (Messick, 1993), which has proven to be implemented in human decision making and highly efficient as a portfolio strategy (DeMiguel, Garlappi, & Uppal, 2009). In our case, the fact that options are drawn from the same prior (known to the agent) contributes to the optimality of the even allocation. Although the optimal allocation of nonidentically distributed options is not addressed here, this heuristic can be efficient in such situations (Thorngate, 1980). It is important to realize that the optimal low numbers of considered options have been found in the case where their values are not known in advance and come from the same distribution. If agents have strong preferences or have additional information about the expected values of the options (e.g., by sampling them sequentially), then the number of considered alternatives will be further reduced. Of course, if the agents are allowed to sequentially sample options with which they are familiar, a noneven allocation might emerge to be optimal (Callaway et al., 2021; Sepulveda et al., 2020; Tajima et al., 2019). Nonetheless, for binary choice, reward is still maximized at even allocations in sequential sampling when options have not been unevenly sampled in the past (Fudenberg, Strack, & Strzalecki, 2018; Jang et al., 2021). On the other hand, when the number of alternatives is much higher than two, people choose to ignore many of the available options (Thomas, Molter, & Krajbich, 2021), consistent with our findings. Moreover, for fixed‐duration tasks, there is evidence that humans have a choice set of around five in sequential decisions (Reutskaja, Nagel, Camerer, & Rangel, 2011), even if their final allocation might be uneven. This shows once again that a low number of considered options can hardly be taken as evidence of a decisional bottleneck and is more in line with an optimal tradeoff between breadth and depth.

Finally, our results can have important implications for the optimal wiring of neural networks in the brain (Rushworth et al., 2011; Siegel et al., 2015; Vickery et al., 2011; Yoo & Hayden, 2018). First, as just few options should be considered at the same time, it is expected that only those would be encoded in different, albeit possibly overlapping, pools of neurons. Thus, although models consisting of two or three pools that compete for dominance through mutual inhibition can be a sensible idea for binary and ternary decision making (Cisek & Kalaska, 2010; Churchland, Kiani, & Shadlen, 2008; Gold & Shadlen, 2007; Moreno‐Bote, Rinzel, & Rubin, 2007; Roe, Busemeyer, & Townsend, 2001; Usher & McClelland, 2001; Wang, 2008), extrapolating this to many more options (e.g., larger than 10) by splitting neurons into corresponding pools of neurons would be hardly optimal. Our results are, in contrast, consistent with the opposite view that posits that a single pool of neurons is sufficient for decision making (Hayden & Moreno‐Bote, 2018). In this framework, a single pool encodes just one of the available options, the one that is under the focus of attention. Previously attended options produce a background activity against which the current option is compared to, and other options fall outside the representation of the neural network (Hayden & Moreno‐Bote, 2018; Krajbich, Armel, & Rangel, 2010; Lim, O'Doherty, & Rangel, 2011; Redish, 2016; Rich & Wallis, 2016). Thus, comparison and selection between options occurs through a temporal contrast, rather than through mutual inhibition between simultaneously encoded options. This model can be readily extrapolated to multiple many options, with the only dilemma of dividing time or precision into few or many options (like in Fig. 1), thus addressing the associated breadth–depth tradeoffs. The debate of the one‐pool versus several‐pools models remains open (Ballesta & Padoa‐Schioppa, 2019; Hayden & Moreno‐Bote, 2018), but electrophysiology experiments with many options should be able to arbitrate between the two hypotheses under the new computational constraints that we have identified here.

## Conflicts of interest

The authors have no conflicts of interest to declare.

## Data availability

All the numerical work performed to generate the various figures is available as documented Julia code along with a guided notebook at this public Github Repository: https://github.com/jorgeerrz/finite_time_allocation_paper.

## Supporting information

Optimal allocation of finite sampling capacity in accumulator models of multi‐alternative decision making.

## References

[cogs13143-bib-0001] Ballesta, S. , & Padoa‐Schioppa, C. (2019). Economic decisions through circuit inhibition. Current Biology, 29(22), 3814–3824. 31679936 10.1016/j.cub.2019.09.027 PMC6878987

[cogs13143-bib-0002] Beach, L. R. (1993). Broadening the definition of decision making: The role of prechoice screening of options. Psychological Science, 4(4), 215–220.

[cogs13143-bib-0003] Brady, T. F. , Konkle, T. , & Alvarez, G. A. (2011). A review of visual memory capacity: Beyond individual items and toward structured representations. Journal of Vision, 11(5), 4. 10.1167/11.5.4 PMC3405498 21617025

[cogs13143-bib-0004] Busemeyer, J. R. , & Townsend, J. T. (1993). Decision field theory: A dynamic‐cognitive approach to decision making in an uncertain environment. Psychological Review, 100(3), 432. 8356185 10.1037/0033-295x.100.3.432

[cogs13143-bib-0005] Callaway, F. , Rangel, A. , & Griffiths, T. L. (2021). Fixation patterns in simple choice reflect optimal information sampling. PLOS Computational Biology, 17(3).e1008863. 33770069 10.1371/journal.pcbi.1008863 PMC8026028

[cogs13143-bib-0006] Churchland, A. K. , Kiani, R. , & Shadlen, M. N. (2008). Decision‐making with multiple alternatives. Nature Neuroscience, 11(6), 693–702. 18488024 10.1038/nn.2123 PMC2453226

[cogs13143-bib-0007] Cisek, P. , & Kalaska, J. F. (2010). Neural mechanisms for interacting with a world full of action choices. Annual Review of Neuroscience, 33, 269–298. 10.1146/annurev.neuro.051508.135409 20345247

[cogs13143-bib-0008] Cowan, N. , Elliott, E. M. , Saults, J. S. , Morey, C. C. , Mattox, S. , Hismjatullina, A. , & Conway, A. R. (2005). On the capacity of attention: Its estimation and its role in working memory and cognitive aptitudes. Cognitive Psychology, 51(1), 42–100. 16039935 10.1016/j.cogpsych.2004.12.001 PMC2673732

[cogs13143-bib-0009] Del Giudice, M. , & Crespi, B. J. (2018). Basic functional trade‐offs in cognition: An integrative framework. Cognition, 179, 56–70. 29909281 10.1016/j.cognition.2018.06.008

[cogs13143-bib-0010] DeMiguel, V. , Garlappi, L. , & Uppal, R. (2009). Optimal versus naive diversification: How inefficient is the 1/n portfolio strategy? Review of Financial Studies, 22(5), 1915–1953.

[cogs13143-bib-0011] Deutsch, J. A. , & Deutsch, D. (1963). Attention: Some theoretical considerations. Psychological Review, 70(1), 80. 14027390 10.1037/h0039515

[cogs13143-bib-0012] Drugowitsch, J. , Moreno‐Bote, R. , Churchland, A. K. , Shadlen, M. N. , & Pouget, A. (2012). The cost of accumulating evidence in perceptual decision making. Journal of Neuroscience, 32(11), 3612–3628. 22423085 10.1523/JNEUROSCI.4010-11.2012 PMC3329788

[cogs13143-bib-0013] Feng, S. F. , Schwemmer, M. , Gershman, S. J. , & Cohen, J. D. (2014). Multitasking versus multiplexing: Toward a normative account of limitations in the simultaneous execution of control‐demanding behaviors. Cognitive, Affective, & Behavioral Neuroscience, 14(1), 129–146. 10.3758/s13415-013-0236-9 PMC4845905 24481850

[cogs13143-bib-0014] Fischer, R. , & Plessow, F. (2015). Efficient multitasking: Parallel versus serial processing of multiple tasks. Frontiers in Psychology, 6, 1366. 26441742 10.3389/fpsyg.2015.01366 PMC4561751

[cogs13143-bib-0015] Fletcher, R. (2013). Practical methods of optimization. John Wiley & Sons.

[cogs13143-bib-0016] Fudenberg, D. , Strack, P. , & Strzalecki, T. (2018). Speed, accuracy, and the optimal timing of choices. American Economic Review, 108(12), 3651–3684.

[cogs13143-bib-0017] Gershman, S. J. , Horvitz, E. J. , & Tenenbaum, J. B. (2015). Computational rationality: A converging paradigm for intelligence in brains, minds, and machines. Science, 349(6245), 273–278. 26185246 10.1126/science.aac6076

[cogs13143-bib-0018] Gigerenzer, G. , & Gaissmaier, W. (2011). Heuristic decision making. Annual Review of Psychology, 62, 451–482. 10.1146/annurev-psych-120709-145346 21126183

[cogs13143-bib-0019] Glöckner, A. , & Betsch, T. (2008). Multiple‐reason decision making based on automatic processing. Journal of Experimental Psychology: Learning, Memory, and Cognition, 34(5), 1055. 18763891 10.1037/0278-7393.34.5.1055

[cogs13143-bib-0020] Gold, J. I. , & Shadlen, M. N. (2007). The neural basis of decision making. Annual Review of Neuroscience, 30.535–574. 10.1146/annurev.neuro.29.051605.113038 17600525

[cogs13143-bib-0021] Griffiths, T. L. , Lieder, F. , & Goodman, N. D. (2015). Rational use of cognitive resources: Levels of analysis between the computational and the algorithmic. Topics in Cognitive Science, 7(2), 217–229. 25898807 10.1111/tops.12142

[cogs13143-bib-0022] Hauser, J. R. , & Wernerfelt, B. (1990). An evaluation cost model of consideration sets. Journal of Consumer Research, 16(4), 393–408.

[cogs13143-bib-0023] Hayden, B. Y. , & Moreno‐Bote, R. (2018). A neuronal theory of sequential economic choice. Brain and Neuroscience Advances, 2.2398212818766675. 32166137 10.1177/2398212818766675 PMC7058205

[cogs13143-bib-0024] Horowitz, E. , & Sahni, S. (1978). Fundamentals of computer algorithms. Computer Science Press.

[cogs13143-bib-0025] Iyengar, S. S. , & Lepper, M. R. (2000). When choice is demotivating: Can one desire too much of a good thing? Journal of Personality and Social Psychology, 79(6), 995. 11138768 10.1037//0022-3514.79.6.995

[cogs13143-bib-0026] Jang, A. I. , Sharma, R. , & Drugowitsch, J. (2021). Optimal policy for attention‐modulated decisions explains human fixation behavior. eLife, 10, e63436. 33769284 10.7554/eLife.63436 PMC8064754

[cogs13143-bib-0027] Krajbich, I. , Armel, C. , & Rangel, A. (2010). Visual fixations and the computation and comparison of value in simple choice. Nature Neuroscience, 13(10), 1292–1298. 20835253 10.1038/nn.2635

[cogs13143-bib-0028] Krajbich, I. , & Rangel, A. (2011). Multialternative drift‐diffusion model predicts the relationship between visual fixations and choice in value‐based decisions. Proceedings of the National Academy of Sciences, 108(33), 13852–13857. 10.1073/pnas.1101328108 PMC3158210 21808009

[cogs13143-bib-0029] Levin, I. P. , Jasper, J. , & Forbes, W. S. (1998). Choosing versus rejecting options at different stages of decision making. Journal of Behavioral Decision Making, 11(3), 193–210.

[cogs13143-bib-0030] Lim, S.‐L. , O'Doherty, J. P. , & Rangel, A. (2011). The decision value computations in the vmFC and striatum use a relative value code that is guided by visual attention. Journal of Neuroscience, 31(37), 13214–13223. 21917804 10.1523/JNEUROSCI.1246-11.2011 PMC6623246

[cogs13143-bib-0031] Luck, S. J. , & Vogel, E. K. (2013). Visual working memory capacity: From psychophysics and neurobiology to individual differences. Trends in Cognitive Sciences, 17(8), 391–400. 23850263 10.1016/j.tics.2013.06.006 PMC3729738

[cogs13143-bib-0032] Ma, W. J. , Husain, M. , & Bays, P. M. (2014). Changing concepts of working memory. Nature Neuroscience, 17(3), 347. 24569831 10.1038/nn.3655 PMC4159388

[cogs13143-bib-0033] Mehta, N. , Rajiv, S. , & Srinivasan, K. (2003). Price uncertainty and consumer search: A structural model of consideration set formation. Marketing Science, 22(1), 58–84.

[cogs13143-bib-0034] Messick, D. M. (1993). Equality as a decision heuristic. Barbara Mellers & Jonathan Baron , Psychological perspectives on justice: Theory and applications, 11–31.

[cogs13143-bib-0035] Meyer, D. E. , & Kieras, D. E. (1997). A computational theory of executive cognitive processes and multiple‐task performance: Part I. Basic mechanisms. Psychological Review, 104(1), 3. 9009880 10.1037/0033-295x.104.1.3

[cogs13143-bib-0036] Miller, D. P. (1981). The depth/breadth tradeoff in hierarchical computer menus. In *Proceedings of the Human Factors Society Annual Meeting* (volume 25, pp. 296–300). Los Angeles, CA: SAGE Publications.

[cogs13143-bib-0037] Miller, G. A. (1956). The magical number seven, plus or minus two: Some limits on our capacity for processing information. Psychological Review, 63(2), 81. 13310704

[cogs13143-bib-0038] Moreno‐Bote, R. (2010). Decision confidence and uncertainty in diffusion models with partially correlated neuronal integrators. Neural Computation, 22(7), 1786–1811. 20141474 10.1162/neco.2010.12-08-930

[cogs13143-bib-0039] Moreno‐Bote, R. , Ramírez‐Ruiz, J. , Drugowitsch, J. , & Hayden, B. Y. (2020). Heuristics and optimal solutions to the breadth–depth dilemma. Proceedings of the National Academy of Sciences, 117(33), 19799–19808. 10.1073/pnas.2004929117 PMC7443877 32759219

[cogs13143-bib-0040] Moreno‐Bote, R. , Rinzel, J. , & Rubin, N. (2007). Noise‐induced alternations in an attractor network model of perceptual bistability. Journal of Neurophysiology, 98(3), 1125–1139. 17615138 10.1152/jn.00116.2007 PMC2702529

[cogs13143-bib-0041] Norman, D. A. , & Shallice, T. (1986). Attention to action. In Richard J. Davidson , Gary E. Schwartz & David Shapiro , Consciousness and self‐regulation (pp. 1–18). Springer.

[cogs13143-bib-0042] Olshavsky, R. W. (1979). Task complexity and contingent processing in decision making: A replication and extension. Organizational Behavior and Human Performance, 24(3), 300–316.

[cogs13143-bib-0043] Payne, J. W. (1976). Task complexity and contingent processing in decision making: An information search and protocol analysis. Organizational Behavior and Human Performance, 16(2), 366–387.

[cogs13143-bib-0044] Ratcliff, R. , & Murdock, B. B. (1976). Retrieval processes in recognition memory. Psychological Review, 83(3), 190.

[cogs13143-bib-0045] Ratcliff, R. , & Smith, P. L. (2004). A comparison of sequential sampling models for two‐choice reaction time. Psychological Review, 111(2), 333. 15065913 10.1037/0033-295X.111.2.333 PMC1440925

[cogs13143-bib-0046] Redish, A. D. (2016). Vicarious trial and error. Nature Reviews Neuroscience, 17(3), 147. 26891625 10.1038/nrn.2015.30 PMC5029271

[cogs13143-bib-0047] Reutskaja, E. , Nagel, R. , Camerer, C. F. , & Rangel, A. (2011). Search dynamics in consumer choice under time pressure: An eye‐tracking study. American Economic Review, 101(2), 900–926.

[cogs13143-bib-0048] Rich, E. L. , & Wallis, J. D. (2016). Decoding subjective decisions from orbitofrontal cortex. Nature Neuroscience, 19(7), 973–980. 27273768 10.1038/nn.4320 PMC4925198

[cogs13143-bib-0049] Roberts, J. H. , & Lattin, J. M. (1991). Development and testing of a model of consideration set composition. Journal of Marketing Research, 28(4), 429–440.

[cogs13143-bib-0050] Roe, R. M. , Busemeyer, J. R. , & Townsend, J. T. (2001). Multialternative decision field theory: A dynamic connectionst model of decision making. Psychological Review, 108(2), 370. 11381834 10.1037/0033-295x.108.2.370

[cogs13143-bib-0051] Rushworth, M. F. , Noonan, M. P. , Boorman, E. D. , Walton, M. E. , & Behrens, T. E. (2011). Frontal cortex and reward‐guided learning and decision‐making. Neuron, 70(6), 1054–1069. 21689594 10.1016/j.neuron.2011.05.014

[cogs13143-bib-0052] Russell, S. , & Wefald, E. (1991). Principles of metareasoning. Artificial Intelligence, 49(1–3), 361–395.

[cogs13143-bib-0053] Russell, S. J. , & Subramanian, D. (1994). Provably bounded‐optimal agents. Journal of Artificial Intelligence Research, 2, 575–609.

[cogs13143-bib-0054] Scheibehenne, B. , Greifeneder, R. , & Todd, P. M. (2010). Can there ever be too many options? A meta‐analytic review of choice overload. Journal of Consumer Research, 37(3), 409–425.

[cogs13143-bib-0055] Sepulveda, P. , Usher, M. , Davies, N. , Benson, A. A. , Ortoleva, P. , & De Martino, B. (2020). Visual attention modulates the integration of goal‐relevant evidence and not value. eLife, 9, e60705. 33200982 10.7554/eLife.60705 PMC7723413

[cogs13143-bib-0056] Shenhav, A. , Musslick, S. , Lieder, F. , Kool, W. , Griffiths, T. L. , Cohen, J. D. , & Botvinick, M. M. (2017). Toward a rational and mechanistic account of mental effort. Annual Review of Neuroscience, 40, 99–124. 10.1146/annurev-neuro-072116-031526 28375769

[cogs13143-bib-0057] Shocker, A. D. , Ben‐Akiva, M. , Boccara, B. , & Nedungadi, P. (1991). Consideration set influences on consumer decision‐making and choice: Issues, models, and suggestions. Marketing Letters, 2(3), 181–197.

[cogs13143-bib-0058] Siegel, M. , Buschman, T. J. , & Miller, E. K. (2015). Cortical information flow during flexible sensorimotor decisions. Science, 348(6241), 1352–1355. 26089513 10.1126/science.aab0551 PMC4721574

[cogs13143-bib-0059] Simon, H. A. (1972). Theories of bounded rationality. Decision and Organization, 1(1), 161–176.

[cogs13143-bib-0060] Sleezer, B. J. , Castagno, M. D. , & Hayden, B. Y. (2016). Rule encoding in orbitofrontal cortex and striatum guides selection. Journal of Neuroscience, 36(44), 11223–11237. 27807165 10.1523/JNEUROSCI.1766-16.2016 PMC5148240

[cogs13143-bib-0061] Stigler, G. J. (1961). The economics of information. Journal of Political Economy, 69(3), 213–225.

[cogs13143-bib-0062] Tajima, S. , Drugowitsch, J. , Patel, N. , & Pouget, A. (2019). Optimal policy for multi‐alternative decisions. Nature Neuroscience, 22(9), 1503–1511. 31384015 10.1038/s41593-019-0453-9

[cogs13143-bib-0063] Thomas, A. W. , Molter, F. , & Krajbich, I. (2021). Uncovering the computational mechanisms underlying many‐alternative choice. eLife, 10, e57012. 33821787 10.7554/eLife.57012 PMC8025657

[cogs13143-bib-0064] Thorngate, W. (1980). Efficient decision heuristics. Behavioral Science, 25(3), 219–225.

[cogs13143-bib-0065] Treisman, A. (1998). Feature binding, attention and object perception. Philosophical Transactions of the Royal Society of London. Series B: Biological Sciences, 353(1373), 1295–1306. 9770223 10.1098/rstb.1998.0284 PMC1692340

[cogs13143-bib-0066] Treisman, A. M. (1969). Strategies and models of selective attention. Psychological Review, 76(3), 282. 4893203 10.1037/h0027242

[cogs13143-bib-0067] Usher, M. , & McClelland, J. L. (2001). The time course of perceptual choice: The leaky, competing accumulator model. Psychological Review, 108(3), 550. 11488378 10.1037/0033-295x.108.3.550

[cogs13143-bib-0068] Vickery, T. J. , Chun, M. M. , & Lee, D. (2011). Ubiquity and specificity of reinforcement signals throughout the human brain. Neuron, 72(1), 166–177. 21982377 10.1016/j.neuron.2011.08.011

[cogs13143-bib-0069] Vul, E. , Goodman, N. , Griffiths, T. L. , & Tenenbaum, J. B. (2014). One and done? Optimal decisions from very few samples. Cognitive Science, 38(4), 599–637. 24467492 10.1111/cogs.12101

[cogs13143-bib-0070] Wang, X.‐J. (2008). Decision making in recurrent neuronal circuits. Neuron, 60(2), 215–234. 18957215 10.1016/j.neuron.2008.09.034 PMC2710297

[cogs13143-bib-0071] Yantis, S. , & Johnston, J. C. (1990). On the locus of visual selection: Evidence from focused attention tasks. Journal of Experimental Psychology: Human Perception and Performance, 16(1), 135. 2137515 10.1037//0096-1523.16.1.135

[cogs13143-bib-0072] Yoo, S. B. M. , & Hayden, B. Y. (2018). Economic choice as an untangling of options into actions. Neuron, 99(3), 434–447. 30092213 10.1016/j.neuron.2018.06.038 PMC6280664

[cogs13143-bib-0073] Zylberberg, A. , Dehaene, S. , Roelfsema, P. R. , & Sigman, M. (2011). The human turing machine: A neural framework for mental programs. Trends in Cognitive Sciences, 15(7), 293–300. 21696998 10.1016/j.tics.2011.05.007

