# Supplementary material for: Optimal Allocation of Finite Sampling Capacity in Accumulator Models of Multialternative Decision Making
Source: Cogn Sci. 2022 May 6;46(5):e13143. doi: 10.1111/cogs.13143 (PMC9285422; doi:10.1111/cogs.13143)
Supplement: Supplementary file 1 — Optimal allocation of finite sampling capacity in accumulator models of multi‐alternative decision making. [file COGS-46-0-s001.pdf]

Appendix for "Optimal allocation of finite sampling capacity in accumulator models of multi-alternative decision making"

Jorge Ramírez-Ruiz[1] and Rubén Moreno-Bote[1,2]

[1]Center for Brain and Cognition, and Department of Information and Communication Technologies, Universitat Pompeu Fabra, Barcelona, Spain
[2]Serra Húnter Fellow Programme, Universitat Pompeu Fabra, Barcelona, Spain

A Mathematical details

Comments and mathematical proofs supporting claims in the main manuscript. All cross-references to equations in this appendix are preceded by A, e.g. Eq. (A.2). Otherwise, cross-references to main manuscript are as usual.

A.1 Expected square distance between two random i.i.d. points

The expected square distance between two independently and identically distributed random points $x, y \sim p(\cdot)$ is, by the law of the unconscious statistician,

$$\begin{aligned}\mathbb{E}[d^2(x,y)] &= \int (x-y)^2 p(x,y)\,\mathrm{d}x\,\mathrm{d}y = \int x^2 p(x)\,\mathrm{d}x + \int y^2 p(y)\,\mathrm{d}y - 2\int x\,p(x)\,\mathrm{d}x \int y\,p(y)\,\mathrm{d}y \\ &= 2\mathbb{E}[x^2] - 2\mathbb{E}[x]^2 \\ &= 2\mathrm{Var}[x]\end{aligned}$$

A.2 Posterior mean of drift is a monotonously increasing function of accumulated evidence

Here we prove that the posterior mean of the drift, which is a random variable with probability distribution defined by Bayes' rule, Eq. (3), is a monotonously increasing function of evidence x. We have seen that the expected value of a drift μ given the accumulated evidence x, for any option (and thus here dropping indices) is given by

$$\hat{\mu}(x,t,\sigma,\theta) = \frac{\int \mathrm{d}\mu\,\mu \mathcal{N}(x|\mu t, \sigma^2 t) p_\theta(\mu)}{p(x|t,\sigma,\theta)}, \tag{A.1}$$

where $p_\theta(\mu)$ is the prior probability of the drifts, with hyperparameters θ and $p(x|t,\sigma,\theta)$ is the marginalized probability distribution of the evidence. To know if $\hat{\mu}(x,t,\sigma,\theta)$ is an increasing function of x, we simply derive, and we expect the derivative to be always positive,

$$\begin{aligned}\frac{\mathrm{d}\hat{\mu}}{\mathrm{d}x} =& \frac{p(x|t,\sigma,\theta)\int \mathrm{d}\mu\,\mu\left(-\frac{x-\mu t}{\sigma^2 t}\right)\mathcal{N}(x|\mu t,\sigma^2 t)p_\theta(\mu)}{p(x|t,\sigma,\theta)^2} \\ &- \frac{\int \mathrm{d}\mu\,\mu\mathcal{N}(x|\mu t,\sigma^2 t)p_\theta(\mu)\int \mathrm{d}\mu\left(-\frac{x-\mu t}{\sigma^2 t}\right)\mathcal{N}(x|\mu t,\sigma^2 t)p_\theta(\mu)}{p(x|t,\sigma,\theta)^2} > 0 \\ \iff& \frac{1}{\sigma^2}\left(\mathbb{E}\left[\mu^2|x,t,\sigma,\theta\right] - \mathbb{E}\left[\mu|x,t,\sigma,\theta\right]^2\right) = \frac{\mathrm{Var}\left[p(\mu|x,t,\sigma,\theta)\right]}{\sigma^2} > 0,\end{aligned}$$

where

$$\mathbb{E}\left[\mu^n|x,t,\sigma,\theta\right] = \frac{\int \mathrm{d}\mu\, \mu^n \mathcal{N}(x|\mu t, \sigma^2 t) p_\theta(\mu)}{\int \mathrm{d}\mu\, \mathcal{N}(x|\mu t, \sigma^2 t) p_\theta(\mu)}.$$

Since the variance is the expected value of a positive quantity, then we conclude that the expected value of the drift is a monotonously increasing function of the observed accumulated evidence x for any prior.

A.3 Expected value of the drift in the small capacity limit

Here we show that in the small capacity limit, the utility in Eq. (9) can be written as in Eq. (11) for any regular prior distribution. Our strategy is to study the limiting behaviors of the cumulative density function (described below in Sec. A.4) and the posterior mean of the drift (detailed in this section) that appear in Eq. (9) as $C = \frac{\sigma_0^2}{\sigma^2}T$ goes to zero.

From Bayes's rule, Eq. (3), the posterior mean of the drift is given by

$$\hat{\mu}(x,t,\sigma,\theta) = \frac{1}{\sqrt{2\pi\sigma^2 t}} \frac{\int \mathrm{d}\mu\, \mu\, \exp\left(-\frac{1}{2\sigma^2 t}(\mu t - x)^2\right) p_\theta(\mu|\theta)}{p(x|t,\sigma,\theta)}. \tag{A.2}$$

Let us focus on the numerator, which we will interpret as the expectation value of $\mu\, \exp\left(-\frac{1}{2\sigma^2 t}(\mu t - x)^2\right)$ with respect to the prior. We assume the prior to be such that this expectation is finite for all x and that all its moments are finite (e.g, Gaussian and uniform distributions). We define $z \equiv z(x) \equiv \frac{1}{\sqrt{\sigma^2 t}}(x - \mu_0 t)$ and $\mu_s \equiv \frac{1}{\sqrt{\sigma^2 t}}(\mu t - \mu_0 t)$, and by adding and subtracting $\mu_0 t$ at the exponent, we can write the numerator in the above equation as

$$\begin{aligned}
\mathbb{E}_\theta\left[\mu\, \exp\left(-\frac{1}{2\sigma^2 t}(\mu t - x)^2\right)\right] &= \mathbb{E}_\theta\left[\mu\, \exp\left(-\frac{1}{2\sigma^2 t}(\mu t - \mu_0 t + \mu_0 t - x)^2\right)\right] \\
&= \exp\left(-\frac{z^2}{2}\right)\mathbb{E}_\theta\left[\mu\, \exp\left(z\mu_s - \frac{1}{2}\mu_s^2\right)\right] \\
&= \exp\left(-\frac{z^2}{2}\right)\left\{\mathbb{E}_\theta\left[\mu_0\, \exp\left(z\mu_s - \frac{1}{2}\mu_s^2\right)\right]\right. \\
&\quad \left. + \mathbb{E}_\theta\left[\sqrt{\frac{\sigma^2}{t}}\mu_s\, \exp\left(z\mu_s - \frac{1}{2}\mu_s^2\right)\right]\right\}.
\end{aligned}$$

Next, we note that the exponential in the expectations is the generating function of the Hermite polynomials, and thus

$$\exp\left(z\mu_s - \frac{1}{2}\mu_s^2\right) = \sum_{n=0}^{\infty} \mathrm{He}_n(z)\frac{\mu_s^n}{n!}.$$

By replacing the exponential with the infinite series in the above expectation, Eq. (A.2), we obtain

$$\begin{aligned}
p(x|t,\sigma,\theta)\hat{\mu}(x,t,\sigma,\theta) =& \frac{\exp\left(-\frac{z^2}{2}\right)}{\sqrt{2\pi\sigma^2 t}}\left\{\mathbb{E}_\theta\left[\mu_0 \sum_{n=0}^{\infty}\mathrm{He}_n(z)\frac{\mu_s^n}{n!}\right] + \mathbb{E}_\theta\left[\sqrt{\frac{\sigma^2}{t}}\mu_s \sum_{n=0}^{\infty}\mathrm{He}_n(z)\frac{\mu_s^n}{n!}\right]\right\} \\
=& \frac{\mathcal{N}(z|0,1)}{\sqrt{\sigma^2 t}}\left\{\sum_{n=0}^{\infty}\frac{1}{n!}\mathbb{E}_\theta\left[\mu_s^n\right]\mu_0 \mathrm{He}_n(z) + \sum_{n=0}^{\infty}\frac{1}{n!}\mathbb{E}_\theta\left[\mu_s^{n+1}\right]\sqrt{\frac{\sigma^2}{t}}\mathrm{He}_n(z)\right\} \\
=& \frac{\mathcal{N}(z|0,1)}{\sqrt{\sigma^2 t}}\left\{\sum_{n=0}^{\infty}\frac{1}{n!}\mathbb{E}_\theta\left[\frac{(\mu-\mu_0)^n}{\sqrt{\sigma^2/t}^n}\right]\left(\mu_0\mathrm{He}_n(z) + \sqrt{\frac{\sigma^2}{t}}n\mathrm{He}_{n-1}(z)\right)\right\} \\
=& \frac{\mathcal{N}(z|0,1)}{\sqrt{\sigma^2 t}}\left\{\sum_{n=0}^{\infty}\frac{1}{n!}\sqrt{\frac{C}{M}}^{n-1}\mathbb{E}_\theta\left[\frac{(\mu-\mu_0)^n}{\sigma_0^n}\right]\right. \\
& \left. \times\left(\sqrt{\frac{C}{M}}\mu_0\mathrm{He}_n(z) + \sigma_0 n\mathrm{He}_{n-1}(z)\right)\right\},
\end{aligned}$$

where we have used that all the moments of the prior are finite and the sum is well defined. Note that to obtain the third line we have shifted the second index $n+1 \to n$ and used that the term $n\mathrm{He}_{n-1}(z)$ is zero for $n=0$.

We now insert the above series into the expression of utility in Eq. (9) to obtain

$$\begin{aligned}
\hat{U}(t,\sigma,\theta) &= \int \mathrm{d}x\, \frac{\mathrm{d}}{\mathrm{d}x}\left\{[F_x(x|t,\sigma,\theta)]^M\right\}\hat{\mu}(x,t,\sigma,\theta) \\
&= \int \mathrm{d}x\; M\,[F_x(x|t,\sigma,\theta)]^{M-1}\,\frac{\mathcal{N}(z|0,1)}{\sqrt{\sigma^2 t}} \\
&\quad\times \left\{\sum_{n=0}^{\infty}\frac{1}{n!}\sqrt{\frac{C}{M}}^{\,n-1}\mathbb{E}_\theta\left[\frac{(\mu-\mu_0)^n}{\sigma_0^n}\right]\left(\sqrt{\frac{C}{M}}\mu_0\mathrm{He}_n(z)+\sigma_0 n\mathrm{He}_{n-1}(z)\right)\right\} \\
&= \int \mathrm{d}z\; M\,[F_z(z|t,\sigma,\theta)]^{M-1}\mathcal{N}(z|0,1)\left[\mu_0+\sqrt{\frac{C}{M}}\sigma_0 z\right]+\mathcal{O}(C),
\end{aligned}$$

where in the second line it is implicit that z depends on x, and in the last line we have made a linear transformation of variables from x to $z=z(x)$. We also note that as the integral in the last line only involves polynomials in z that are weighted by the standard normal (and by a cumulative, which is bounded to be in the range $[0,1]$), their integrals are finite, and thus we can truncate the series at the first leading order, which is order $\sqrt{C}$. It remains to see whether the cumulative density function $F_z(z|t,\sigma,\theta)$ contributes order $\sqrt{C}$ or larger, and we show below in Sec. (A.4) that the former is actually true, such that $F_z(z|t,\sigma,\theta)=\frac{1}{2}\left[1+\mathrm{erf}\left(\frac{z}{\sqrt{2}}\right)\right]+\mathcal{O}(C)$. With all this, we can approximate the utility up to order $\sqrt{C}$ as

$$\hat{U}(C,M,\mu_0)=\mu_0+\sigma_0\sqrt{C}\sqrt{M}\int_{-\infty}^{\infty}\mathrm{d}z\left[\frac{1}{2}\left(1+\mathrm{erf}\left(\frac{z}{\sqrt{2}}\right)\right)\right]^{M-1}\mathcal{N}(z|0,1)\,z+\mathcal{O}(C), \qquad \text{(A.3)}$$

which is identical to Eq. (11) in main manuscript.

A.4 Distribution of evidence at small capacity limit

Here, we find an approximation to the marginalized probability distribution of the evidence at small capacity. From Bayes' rule and the law of the unconscious statistician,

$$p(x|t,\sigma,\theta)=\int \mathrm{d}\mu\,\mathcal{N}(x|\mu t,\sigma^2 t)p_\theta(\mu|\theta)=\mathbb{E}_\theta\left[\mathcal{N}(x|\mu t,\sigma^2 t)\right].$$

To compute this expectation, we follow the same procedure as in Sec. A.3. We define $z\equiv\frac{1}{\sqrt{\sigma^2 t}}(x-\mu_0 t)$ and $\mu_s\equiv\frac{1}{\sqrt{\sigma^2 t}}(\mu t-\mu_0 t)$ and add and subtract $\mu_0 t$ at the exponent, to obtain

$$\begin{aligned}
p(x|t,\sigma,\theta) &= \mathbb{E}_\theta\left[\exp\left(-\frac{1}{2\sigma^2 t}(x-\mu t)^2\right)\right] \\
&= \mathbb{E}_\theta\left[\exp\left(-\frac{1}{2\sigma^2 t}(x-\mu_0 t+\mu_0 t-\mu t)^2\right)\right] \\
&= \exp\left(-\frac{z^2}{2}\right)\mathbb{E}_\theta\left[\exp\left(z\mu_s-\frac{1}{2}\mu_s^2\right)\right].
\end{aligned}$$

Next, we again identify the exponential generating function of the Hermite polynomials,

$$\exp\left(z\mu_s-\frac{1}{2}\mu_s^2\right)=\sum_{n=0}^{\infty}\mathrm{He}_n(z)\frac{\mu_s^n}{n!},$$

and thus we obtain a series for the probability distribution of the evidence,

$$
\begin{aligned}
p(x|t,\sigma,\theta) =& \frac{1}{\sqrt{2\pi\sigma^2 t}} \exp\left(-\frac{z^2}{2}\right) \mathbb{E}_\theta \left[\sum_{n=0}^{\infty} \mathrm{He}_n(z) \frac{\mu_s^n}{n!}\right] \\
=& \frac{\exp\left(-\frac{z^2}{2}\right)}{\sqrt{2\pi\sigma^2 t}} \sum_{n=0}^{\infty} \frac{1}{n!} \mathbb{E}_\theta \left[\mu_s^n\right] \mathrm{He}_n(z) \\
=& \frac{\exp\left(-\frac{z^2}{2}\right)}{\sqrt{2\pi\sigma^2 t}} \sum_{n=0}^{\infty} \frac{1}{n!} \mathbb{E}_\theta \left[\frac{(\mu-\mu_0)^n}{\sqrt{\sigma^2/t}^n}\right] \mathrm{He}_n(z) \\
=& \frac{\exp\left(-\frac{z^2}{2}\right)}{\sqrt{2\pi\sigma^2 t}} \sum_{n=0}^{\infty} \frac{1}{n!} \sqrt{\frac{C}{M}}^n \mathbb{E}_\theta \left[\frac{(\mu-\mu_0)^n}{\sigma_0^n}\right] \mathrm{He}_n(z).
\end{aligned}
$$

We see that the leading order the distribution of the evidence is a normal distribution, while the order $\sqrt{C}$ is zero. Therefore, its cumulative in the variable $z = z(x)$ is, exactly, up to order $\sqrt{C}$, $F_z(z|t,\sigma,\theta) = \frac{1}{2}\left[1 + \mathrm{erf}\left(\frac{z}{\sqrt{2}}\right)\right] + \mathcal{O}(C)$. This expression has been used in Sec. (A.3).

A.5 Asymptotic limit of relevant integral

In this subsection we want to obtain the asymptotic limit, $M \to \infty$, of the integral

$$
I(M) = \int_{-\infty}^{\infty} \mathrm{d}y \, y \, \frac{\mathrm{d}}{\mathrm{d}y} \Phi^M(y),
$$

appearing in Eqs. (11) and (12), where $\Phi(y)$ is the normal cumulative distribution function,

$$
\Phi(y) = \left(\frac{1}{2} + \frac{1}{2}\,\mathrm{erf}\left(\frac{y}{\sqrt{2}}\right)\right).
$$

Using Extreme Value Theory [1], it can be shown that this cumulative distribution function $\Phi(y)$ belongs to the Gumbel class of the generalized extreme value distributions,

$$
\lim_{M\to\infty} \Phi^M(a_M y + b_M) = G(y),
$$

where $G(y) = \exp(-\exp(-y))$ and

$$
b_M = (2\log(M) - \log(\log(M)) - \log(4\pi))^{1/2} \quad \text{and} \quad a_M = 1/b_M.
$$

Using this result, then our integral develops quite easily,

$$
\begin{aligned}
I(M) \to& \int_{-\infty}^{\infty} \mathrm{d}y \, y \, \frac{\mathrm{d}}{\mathrm{d}y} G\left(\frac{y - b_M}{a_M}\right) \\
=& \int_{-\infty}^{\infty} \mathrm{d}y \left(\frac{y}{b_M} + b_M\right) \frac{\mathrm{d}}{\mathrm{d}y} G(y) \\
=& \frac{1}{b_M} \int_{-\infty}^{\infty} \mathrm{d}y \, y \exp(-y) \exp(-\exp(-y)) + b_M \int_{-\infty}^{\infty} \mathrm{d}y \, \frac{\mathrm{d}}{\mathrm{d}y} G(y) \\
I(M\to\infty) =& \frac{\gamma}{b_M} + b_M,
\end{aligned}
$$

where $\gamma \approx 0.577$ is Euler's constant.

A.6 Expected utility for uniform prior

For this choice of prior, drifts are all drawn independently and identically from a uniform probability distribution between zero and one. That is,

$$p(\mu_i) = \Theta(\mu_i)\Theta(1-\mu_i),$$

where $\Theta(x)$ is the Heaviside step function. We can substitute this prior into eq. (3) to obtain the posterior probability distribution for the drifts,

$$p(\mu_i|x_i,\sigma,t_i,\theta) = \begin{cases} \frac{\mathcal{N}\left(\mu_i|\frac{x_i}{t_i},\frac{\sigma^2}{t_i}\right)}{\int_0^1 \mathcal{N}\left(\mu_i|\frac{x_i}{t_i},\frac{\sigma^2}{t_i}\right)\mathrm{d}\mu_i} & \mu_i \in [0,1] \\ 0 & \text{otherwise.} \end{cases}$$

This will produce an expectation value for each drift,

$$\hat{\mu}_i(x_i,t_i,\sigma) \equiv \mathbb{E}\left[\mu_i|x_i,t_i,\sigma\right] = \frac{x_i}{t_i} + \frac{\sigma}{\sqrt{2\pi t_i}} \frac{\exp\left(-\frac{x_i^2}{2\sigma^2 t_i}\right) - \exp\left(-\frac{(x_i-t_i)^2}{2\sigma^2 t_i}\right)}{\frac{1}{2}\left[\operatorname{erf}\left(\frac{x_i}{\sqrt{2\sigma^2 t_i}}\right) - \operatorname{erf}\left(\frac{x_i-t_i}{\sqrt{2\sigma^2 t_i}}\right)\right]}, \tag{A.4}$$

where the denominator is related to the probability distribution of the evidence x_i, which we can find by marginalizing over drifts,

$$\begin{aligned} p(x_i|t_i,\sigma) &= \int_0^1 \mathrm{d}\mu \frac{1}{\sqrt{2\pi\sigma^2 t_i}} \exp\left(-\frac{1}{2\sigma^2 t_i}(x_i-\mu_i t_i)^2\right) \\ &= \frac{1}{2t_i}\left[\operatorname{erf}\left(\frac{x_i}{\sqrt{2\sigma^2 t_i}}\right) - \operatorname{erf}\left(\frac{x_i-t_i}{\sqrt{2\sigma^2 t_i}}\right)\right]. \end{aligned} \tag{A.5}$$

We will use from now on the assumption of even time allocation, $t_i = t = \frac{T}{M}$ for all i. The cumulative probability distribution for the evidence in Eq. (A.5) is, integrating by parts,

$$\begin{aligned} F(x|t,\sigma) &= \int_{-\infty}^{x} p(x'|t,\sigma)\,\mathrm{d}x' \\ &= \frac{1}{2}\left(1+\operatorname{erf}\left(\frac{x-t}{\sqrt{2\sigma^2 t}}\right)\right) + \frac{x}{2t}\left(\operatorname{erf}\left(\frac{x}{\sqrt{2\sigma^2 t}}\right) - \operatorname{erf}\left(\frac{x-t}{\sqrt{2\sigma^2 t}}\right)\right) \\ &+ \sqrt{\frac{\sigma^2}{2\pi t}}\left[\exp\left(-\frac{x^2}{2\sigma^2 t}\right) - \exp\left(-\frac{(x-t)^2}{2\sigma^2 t}\right)\right] \\ &= \frac{1}{2}\left[1+\operatorname{erf}\left(\frac{x-t}{\sqrt{2\sigma^2 t}}\right)\right] + tp(x|t,\sigma)\hat{\mu}(x,t,\sigma), \end{aligned}$$

where in the last equality we have rewritten the solution in a convenient form. Hence, the product of the expected value with the probability density can be rewritten in terms of the cumulative function, from the previous equation,

$$\hat{\mu}(x,t,\sigma)p(x|t,\sigma) = \frac{1}{t}F(x|t,\sigma) - \frac{1}{2t}\left[1+\operatorname{erf}\left(\frac{x-t}{\sqrt{2\sigma^2 t}}\right)\right],$$

and using eq. (9) we get the expression for the utility,

$$\hat{U}(M,t,\sigma) = \frac{M}{t}\int_{-\infty}^{\infty} \mathrm{d}x\,\left[F(x|t,\sigma)\right]^{M-1}\left\{F(x|t,\sigma) - \frac{1}{2}\left[1+\operatorname{erf}\left(\frac{x-t}{\sqrt{2\sigma^2 t}}\right)\right]\right\}. \tag{A.6}$$

A.7 Expected utility for Gaussian bimodal prior

The expected utility for the bimodal Gaussian prior with modes μ_1 and μ_2, each with a variance σ_0^2, is quite similar to the unimodal, Eq. (10), and follows the straightforward application of Eq. (9). The probability distribution of the evidence marginalized over drifts is $p(x|t,\sigma,\theta) = \frac{1}{2}\mathcal{N}(x|\mu_1 t, \sigma^2 t+\sigma_0^2 t^2) + \frac{1}{2}\mathcal{N}(x|\mu_2 t, \sigma^2 t + \sigma_0^2 t^2)$. Therefore the cumulative is

$$F(x|t,\sigma,\theta) = \frac{1}{2}\Phi(x|\mu_1 t, \sigma^2 t + \sigma_0^2 t^2) + \frac{1}{2}\Phi(x|\mu_2 t, \sigma^2 t + \sigma_0^2 t^2),$$

where $\Phi(x|\mu_m, \sigma_m^2)$ is the normal cumulative distribution for one mode. However, the expected value of the drift is a bit more involved, since the posterior distribution over drifts takes a different form,

$$p(\mu|x,t,\sigma,\theta) = \frac{\sigma_t}{\sqrt{2\pi\sigma^2 t\sigma_0^2}\, p(x|t,\sigma,\theta)} \sum_i \frac{1}{2}\mathcal{N}(\mu|\hat{\mu}_i, \sigma_t^2) \exp\left(-\frac{1}{2(\sigma_0^2 t^2 + \sigma^2 t)}\left(\mu_i t - x\right)^2\right),$$

where $1/\sigma_t^2 = t/\sigma^2 + 1/\sigma_0^2$ and

$$\hat{\mu}_i = \frac{\sigma_t^2}{\sigma_0^2}\mu_i + \frac{\sigma_t^2}{\sigma^2}x.$$

Consequently, the expected value will be

$$\hat{\mu}(x,t,\sigma,\theta) = \frac{1}{\sqrt{2\pi(\sigma_0^2 t^2 + \sigma^2 t)}}\frac{1}{p(x|t,\sigma,\theta)} \sum_i \frac{\hat{\mu}_i}{2} \exp\left(-\frac{1}{2(\sigma_0^2 t^2 + \sigma^2 t)}\left(\mu_i t - x\right)^2\right).$$

Then, expected utility is

$$\hat{U}(M,t,\sigma,\theta) = M\int_{-\infty}^{\infty} \mathrm{d}x\, F(x|t,\sigma,\theta)^{M-1} \sum_i \frac{\hat{\mu}_i}{2}\mathcal{N}\left(x|\mu_i t, \sigma_0^2 t^2 + \sigma^2 t\right). \qquad \text{(A.7)}$$

This expression is numerically integrated and used in Fig. 4.

A.8 Stochastic gradient ascent method for Gaussian prior

To maximize utility, Eq. (14) in main manuscript, under the time constraint, we can make use of unconstrained optimization through Lagrangian multipliers. We construct the Lagrangian given by

$$L(\mathbf{t},\theta,\lambda) = \hat{U}(\mathbf{t},\sigma,\theta) + \lambda h(\mathbf{t}) + \boldsymbol{\mu}\cdot\mathbf{g}(\mathbf{t}), \qquad \text{(A.8)}$$

where $h(\mathbf{t}) = \sum_{i=1}^M t_i - T$ is the equality constraint that defines the hyperplane and $0 \leq g_i(\mathbf{t}) = t_i$ is the inequality constraint forcing all times i to be non-negative and thus defining the simplex. The quantities λ and $\boldsymbol{\mu}$ are the Lagrangian multipliers. In other words, maximizing utility, Eq. (14), subject to the initial constraints can be done by optimizing the Lagrangian, Eq. (A.8), with respect to $\mathbf{t}$, λ and $\boldsymbol{\mu}$ subject to Karush-Kuhn-Tucker conditions [2]

$$g_i(\mathbf{t}) \geq 0, \quad \text{for all } i \qquad \text{(A.9a)}$$

$$\mu_j \geq 0, \quad \text{for all } j \qquad \text{(A.9b)}$$

$$\boldsymbol{\mu}\cdot\mathbf{g}(\mathbf{t}) = 0 \qquad \text{(A.9c)}$$

We notice that the first two conditions imply that the third can be rewritten as $\mu_i t_i = 0$ for all i. By optimizing the Lagrangian, Eq. (A.8), we obtain the following system of equations

$$\nabla_{\mathbf{t}}\hat{U}(\mathbf{t}^*,\sigma,\theta) + \lambda^*\mathbf{1} + \boldsymbol{\mu}^* = \mathbf{0}, \qquad \text{(A.10)}$$

where $\mathbf{t}^*, \lambda^*, \boldsymbol{\mu}^*$ denote the critical points of the Lagrangian. We note that the symmetrical point in M active dimensions, denoted by $\mathbf{t}_M^e$, where $t_{M,i}^e = T/M$ for $i = 1,...,M$, is a critical point of the

Lagrangian. This is because the partial derivatives with respect to the utility have to be equal at $\mathbf{t}_M^e$, and since this point lies in the interior of the $(M-1)$-simplex, the $\boldsymbol{\mu}_i^e = 0$ for $i = 1, \ldots, M$. Therefore $\mathbf{t}_M^e$ complies with Eq. (A.10) and is indeed a critical point.

Next, we detail the gradient ascent method used to obtain Fig. 5**e**. As explained above, we want to optimize utility, Eq. (14), subject to a set of equality, Eq. (1), and inequality constraints, $t_i \geq 0$, as described in section "Even allocation is optimal" of the Results. As all our constraints are linear, we can make use of the gradient projection method [3]. In this case, we want to obtain the gradient of utility in Eq. (10) and project it in the $(M-1)$-simplex such that the capacity constraint in Eq. (1) is satisfied. Due to the linear capacity equality constraint, this projection is simply given by the linear operator

$$\Pi = \mathbf{Id}_{M\times M} - \frac{1}{M}\mathbf{1}_{M\times M}$$

where $\mathbf{Id}_{M\times M}$ is the $M \times M$ identity matrix and $\mathbf{1}_{M\times M}$ is an $M \times M$ matrix full of ones. Therefore, we can maximize utility by updating $\mathbf{t}^{(k)}$ appropriately,

$$\mathbf{t}^{(k+1)} = \mathbf{t}^{(k)} + \eta\Pi\left(\nabla_{\mathbf{t}}\hat{U}(\mathbf{t}, \sigma, \theta)\right), \tag{A.11}$$

where $\eta = 10^{-1}T$ is the default step size, k is the iteration number, and θ corresponds to the parameters of the Gaussian prior. The utility for an arbitrary time allocation $\mathbf{t}$ for the Gaussian prior case is, using Eq. (14),

$$\hat{\mathbf{U}}(\mathbf{t}, \sigma, \theta) = \mu_0 + \sigma_0 \sum_{i=1}^{N} \int_{-\infty}^{\infty} \mathrm{d}y\, y\, \frac{\exp\left(-\frac{y^2}{2\sigma_i^2}\right)}{\sqrt{2\pi\sigma_i^2}} \prod_{j\neq i} \left\{\frac{1}{2}\left[1 + \mathrm{erf}\left(\frac{y}{\sqrt{2}\sigma_j}\right)\right]\right\}, \tag{A.12}$$

where $\sigma_i^2 = \frac{\sigma_0^2 t_i}{\sigma_0^2 t_i + \sigma^2}$. We can therefore compute the derivative of the previous equation with respect to all components t_i and numerically integrate the expression that results.

In addition to the linear capacity constraint, we have to enforce the inequality constraints as well, i.e. $t_i \geq 0$, which we do by utilizing an active set of constraints. To implement it, we start in a relatively high-dimensional $(M-1)$-simplex, choosing M to be $2M^*$, where M^* is the optimal number of accumulators to sample in the even sampling case (which is estimated before through exploration, see main text). If and whenever any of the components of $\mathbf{t}^{k+1}$ derived from Eq. (A.11) is approaching a border ($t_i^{(k+1)} \approx \tau$ for some i and small τ), the step size decreases until the component effectively reaches zero. In such a case, this dimension is added to the active constraints set (we inactivate the dimension), thus downgrading the simplex to a lower dimension. In this way, our algorithm only reduces the initial dimension of the simplex and never extends it. To initially activate the $2M^*$ dimensions, for any random initial condition $\mathbf{t}_0$, we make sure that all the components are greater than our threshold $t_{0,i} > \tau$ for all $i = 1, ..., 2M^*$.

Finally, in order to avoid potentially getting trapped in local maxima, we add noise at every iteration as follows. At every step k of Eq. (A.11), and with probability $\epsilon = 0.1$, we push the $t_i^{(k)}$ of a randomly chosen dimension i by a magnitude $\delta = 10^{-3}T$ and pull the $t_j^{(k)}$ of another random dimension j in the opposite direction with the same amount in order to stay in the appropriate simplex.

References

[1] Laurens De Haan and Ana Ferreira. *Extreme value theory: an introduction*. Springer Science & Business Media, 2007.

[2] Christopher M Bishop. *Pattern recognition and machine learning*. springer, 2006.

[3] Roger Fletcher. *Practical methods of optimization*. John Wiley & Sons, 2013.
